# Experimental investigation of efficient locomotion of underwater snake robots for lateral undulation and eel-like motion patterns

**DOI:** 10.1186/s40638-015-0029-4

**Published:** 2015-12-14

**Authors:** Eleni Kelasidi, Pål Liljebäck, Kristin Y. Pettersen, Jan T. Gravdahl

**Affiliations:** Centre for Autonomous Marine Operations and Systems, Department of Engineering Cybernetics, NTNU, 7491 Trondheim, Norway; Department of Engineering Cybernetics, NTNU, 7491 Trondheim, Norway

**Keywords:** Underwater snake robots, Modeling of swimming robots, Properties of gait parameters, Energy efficiency

## Abstract

Underwater snake robots offer many interesting capabilities for underwater operations. The long and slender structure of such robots provide superior capabilities for access through narrow openings and within confined areas. This is interesting for inspection and monitoring operations, for instance within the subsea oil and gas industry and within marine archeology. In addition, underwater snake robots can provide both inspection and intervention capabilities and are thus interesting candidates for the next generation inspection and intervention AUVs. Furthermore, bioinspired locomotion through oscillatory gaits, like lateral undulation and eel-like motion, is interesting from an energy efficiency point of view. Increasing the motion efficiency in terms of the achieved forward speed by improving the method of propulsion is a key issue for underwater robots. Moreover, energy efficiency is one of the main challenges for long-term autonomy of these systems. In this study, we will consider both these two aspects of efficiency. This paper considers the energy efficiency of swimming snake robots by presenting and experimentally investigating fundamental properties of the velocity and the power consumption of an underwater snake robot for both lateral undulation and eel-like motion patterns. In particular, we investigate the relationship between the parameters of the gait patterns, the forward velocity and the energy consumption for different motion patterns. The simulation and experimental results are seen to support the theoretical findings.

**Electronic supplementary material:**

The online version of this article (doi:10.1186/s40638-015-0029-4) contains supplementary material, which is available to authorized users.

## Background

The use of underwater vehicles has rapidly increased in the last decades since these systems are able to operate in deep and high risk areas which humans can not reach. Nowadays, autonomous underwater vehicles (AUVs) and remotely operated vehicles (ROVs) are widely used in the subsea environment for different challenging tasks [1]. These vehicles are suitable for various work assignments such as inspection, surveillance, maintenance, repairing equipment, building structures, and data collection, and they are extensively used in the subsea oil and gas industry and by the science community. For the long-term autonomy of these systems, energy efficiency is one of the main challenges. In addition, swimming snake robots represent an interesting alternative to conventional ROVs and AUVs. These mechanisms have a long, slender and flexible body which enable them to reach and operate in locations not accessible by larger and more conventional underwater vehicles. At the same time, a swimming snake robot carries manipulation capabilities as an inherent part of its body since it is essentially a mobile manipulator arm. Underwater snake robots thus bring a promising prospective to improve the efficiency and maneuverability of modern-day underwater vehicles [2–5]. A particularly relevant application concerns inspection and maintenance of subsea oil and gas installations, where the ability to reach tight locations in between pipe structures is important. Moreover, for the biological community and marine archeology, snake robots that can swim smoothly with limited noise, and that can navigate in difficult environments such as ship wrecks, are very interesting [2]. To realize operational snake robots for such underwater applications, a number of different control design challenges must first be solved. An important control problem concerns the ability to achieve efficient motion with preferably a minimum amount of consumed energy to be able to undertake longer missions, and this is the topic of this paper.

The majority of previous studies on snake robots considers snake locomotion on ground surfaces, with a multitude of models proposed for this type of snake robots [6]. Empirical and analytic studies of snake locomotion were reported by [7], while the work of [8] is among the first approaches to develop a snake robot prototype. Several land-based snake robots [9–13] and biologically inspired swimming robots [14–29] have been constructed since then. A review of ground snake-like robots can be found in [30, 31]. Comparing amphibious snake robots to the traditional land-based ones, the former have the advantage of adaptability to aquatic environments. The research activity on amphibious snake robots (also referred to as lamprey or eel-like robots) that can operate in aquatic environments, is less extensive. Due to the complex dynamics of swimming snake robots, several different modeling approaches have been carried out in the literature [2–4, 18, 32–40]. A comparison of these approaches is presented in [41]. The majority of previous modeling approaches for underwater snake robots omit the fluid moments (fluid torques) by considering that their effect on the motion of the robot is negligible [34, 36, 42]. However, including the impact of the fluid torques on the power consumption of the system (see e.g. [4]), will improve the accuracy of the model from a hydrodynamic and energy efficiency point of view. The works in [3–5] propose models which consider fluid torques, where drag forces and torques are evaluated numerically. These approaches lack a closed form solution, which is a drawback since a hydrodynamic model in closed form is advantageous for control design and analysis. The works in [2, 43] present a closed form hydrodynamic model, where hydrodynamic forces and torques are considered and where there is no need for algorithmic computation of drag effects. Furthermore, in this approach, both linear and nonlinear drag forces (resistive fluid forces), the added mass effect (reactive fluid forces), the fluid moments, and current effects are considered. The resulting closed form model is well suited for model-based control design schemes and stability analysis. The simulation study in this paper will be based on this model.

In this paper, we will investigate fundamental properties of the velocity dynamics of underwater snake robots that are essential for motion planning purposes and for the efficiency of these systems. Preliminary results were presented in [2, 43–45]. In particular, in [44] a control-oriented model, aimed at control design and stability analysis purposes, of underwater snake robot locomotion was presented. The proposed model in [44], takes into account the added mass effects, the linear drag forces, the torques due to the added mass and linear drag forces, is significantly less complex than the existing models on underwater snake robots, and at the same time has the same essential properties as the complex model presented in [2, 43]. Based on this control-oriented model, an average model of the velocity dynamics was presented in [45]. By using the averaging theory, in [44], fundamental properties are derived regarding the relationship between the gait parameters and the forward velocity. In this paper, we will present a simulation study to investigate the validity of these relationships, and the simulation results will be based on the models presented in [2, 43, 44]. In this paper, we combine the modeling in the preliminary conference papers [2, 43, 44] into a unified presentation and analyze the theoretical findings in [45] in order to address the fundamental properties of locomotion for underwater snake robot. To this end, this paper summarizes the main results of [2, 43–45], to create the foundation which is then used as basis for an experimental validation study and thus present an integrated solution regarding the efficiency of biologically inspired swimming robots.

The main contribution of this paper is the experimental investigation of a set of fundamental properties of the velocity dynamics of underwater snake robots. In [45], the properties were derived based on the control-oriented model of an underwater snake robot proposed in [44]. The derived properties state that the average forward velocity of the robot (1) is a function of the amplitude of the sinusoidal motion pattern, (2) depends on a linear and a nonlinear term of the gait frequency, and (3) depends on the phase shift between the joints. Initially in this paper, we present simulation results to investigate the validity of these properties for both the complex model presented in [2] and the control-oriented model in [44]. Furthermore, in this paper we extend the preliminary results presented in [45] and based on extensive simulation results we show that the forward velocity of the robot: (1) is a class $\mathcal {K}$ function of alpha, i.e., increases when the amplitude of the gait pattern $\alpha$ increases, for small amplitudes, (2) increases almost linearly with respect to the frequency of the gait pattern $\omega$ (i.e., the nonlinear term of $\omega$ has a negligible effect on the achieved forward velocity) and (3) depends on the phase shift between the joints $\delta$. The simulation results show that the derived properties based on the control-oriented model of an underwater snake robot hold also for the complex model where more complex hydrodynamic effects are considered. Then we present experimental results using a physical underwater snake robot [24], and we show that the experimental results support the derived properties of the velocity dynamics. Moreover, the experimental results presented in this paper not only verify the properties regarding the gait parameters of the sinusoidal motion pattern derived based on the control-oriented model and the empirical rules derived based on an extensive simulation study using the complex model, but also validate the control-oriented model as a suitable model for underwater snake robot locomotion. Both the simulation and experimental results are obtained for the two most common swimming patterns for underwater snake robot locomotion: lateral undulation and eel-like motion patterns. To the authors’ best knowledge, an experimental investigation of efficient motion patterns by investigating the relationship between the gait parameters and the forward velocity has not been considered in previous literature.

Another important control problem for underwater vehicles concerns the ability to achieve efficient motion with preferably a minimum amount of consumed energy in order to be able to undertake longer missions. Hence, for the long-term autonomy of underwater vehicles, energy efficiency is one of the main challenges. Preliminary results on this were presented in [46–48]. In particular, in [46], the relationships between the parameters of the gait patterns, the consumed energy, and the forward velocity for different motion patterns for underwater snake robots were investigated. In addition, empirical rules were proposed in order to choose the most efficient motion pattern. In [47], a simulation study was undertaken in order to compare the power consumption of swimming snake robots with that of today’s benchmark solution for subsea inspection, maintenance and repair, which are ROVs. The presented simulation results showed that an underwater snake robot is more energy efficient than a ROV for all the compared motion modes. Furthermore, [48] proposed a multiobjective optimization scheme to obtain optimal gait parameters for underwater snake robots. The proposed optimization method constitutes a general tool to investigate the motion efficiency of different dynamic models of swimming snake robots controlled by sinusoidal motion patterns. To our knowledge, however, no research has been published investigating experimentally the power consumption of underwater snake robots. To this end, in this paper we investigate experimentally the validity of the empirical rules proposed in [46, 47] regarding the relationship between the gait parameters, the velocity and the power consumption. Note that while the derivation of the empirical rules proposed in [46, 47] are based on simulation studies, this paper investigates the validity of the properties through experiments using a physical underwater snake robot [24]. The experimental results are seen to support the empirical rules proposed in [46, 47] regarding the relationship between the gait parameters, the velocity and the power consumption for both lateral undulation and eel-like motion patterns.

The paper is organized as follows “A complex model of underwater snake robots” briefly presents the complex model of an underwater snake robot, while the control-oriented model is outlined in “Control-oriented model of underwater snake robots”. The motion pattern for underwater snake robots and the joint controller are presented in “Joint controller”, followed by a description of the experimental setup in “Experimental setup”. “Relationships between gait parameters, the forward velocity and the power consumption” presents the derived fundamental properties of the velocity dynamics of the robot and the proposed empirical rules regarding the efficient motion of underwater snake robots. Simulation results regarding the relationship between the gait parameters and the forward velocity are presented in “Simulation study: relationships between gait parameters and forward velocity”, followed by an experimental investigation of these properties in “Experimental study: relationships between gait parameters and forward velocity”. “Power consumption of underwater snake robots” presents simulation and experimental results regarding the power consumption of underwater snake robots for both lateral undulation and eel-like motion patterns. Finally, conclusions and suggestions for further research are given in “Conclusions and future work”.

## A complex model of underwater snake robots

The numerical investigation of the fundamental properties of underwater snake robots that is presented in this paper will be based on a model of the kinematics and dynamics of underwater snake robots moving in a virtual horizontal plane. This model was developed in [2, 43], where a detailed description can be found. This section provides a brief presentation of this model.

### Basic notations

**Fig. 1:**
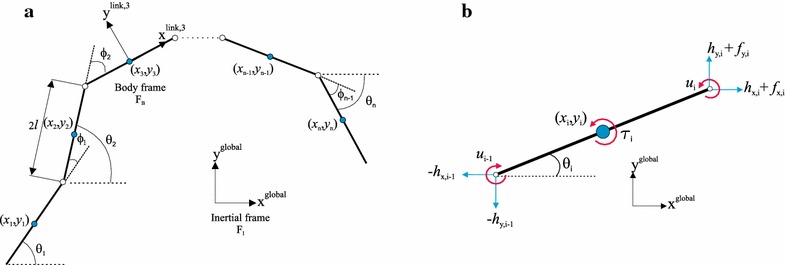
Parameters of the complex model. **a** Kinematic parameters of the robot and **b** forces and torques acting on each link of the robot

The underwater snake robot consists of *n* rigid links of equal length 2*l* interconnected by $n-1$ joints. The links are assumed to have the same mass *m* and moment of inertia $J=\frac{1}{3} m l^{2}$. The mass of each link is uniformly distributed so that the link center of mass (CM) is located at its center point (at length *l* from the joint at each side). The total mass of the snake robot is therefore *nm*. In the following subsections, the kinematics and dynamics of the robot will be described in terms of the mathematical symbols described in Table 1 and illustrated in Fig. 1. The following vectors and matrices are used in the subsequent sections:

$$\begin{aligned} {\mathbf {A}}=\left[ \begin{array}{cccc} 1 &{} 1\\ &{} \ddots &{} \ddots \\ &{} &{} 1 &{} 1 \end{array}\right] ,\quad {\mathbf {D}}=\left[ \begin{array}{ccccc} 1 &{} -1\\ &{} \ddots &{} \ddots \\ &{} &{} 1 &{} -1 \end{array}\right] ~, \end{aligned}$$

$$\begin{aligned} \mathbf{e }=\left[ \begin{array}{ccc} 1&\ldots&1 \end{array}\right] ^{T}\in \mathbb {R}^{n}, \quad {\mathbf {E}} = \left[ \begin{array}{cc} {\mathbf {e}} &{} {\mathbf {0}}_{n\times 1}\\ {\mathbf {0}}_{n\times 1} &{} {\mathbf {e}} \end{array}\right] \in \mathbb {R}^{2n\times 2}\,, \end{aligned}$$

1 $$\begin{aligned} {\mathbf {S}}_{\theta }= & {} \text{ diag }(\sin {{\varvec{\theta }}})\in \mathbb {R}^{{n}\times n},\quad {\mathbf {C}}_{\theta }=\text{ diag }(\cos {{\varvec{\theta }}})\in \mathbb {R}^{{n}\times n}, \\ \dot{{\varvec{\theta }}}^{2}= & {} \left[ \begin{array}{ccc} \dot{\theta _{1}}^{2}&\ldots&\dot{\theta _{n}}^{2} \end{array}\right] ^{T}\in \mathbb {R}^{n},\quad {\mathbf {K}}={\mathbf {A}}^T\left( {\mathbf {D}}{\mathbf {D}}^T\right) ^{-1}{\mathbf {D}},\, \nonumber \end{aligned}$$

where the matrices $\mathbf {A} \in \mathbb {R}^{\left( n-1\right) \times n}$ and $\mathbf {D} \in \mathbb {R}^{\left( n-1\right) \times n}$ represent, respectively, an addition and a difference matrix, which will be used for adding and subtracting pairs of adjacent elements of a vector. Furthermore, the vector $\mathbf {e}$ represents a summation vector, which is used for adding all elements of a *n*-dimensional vector.

**Table 1:** Definition of mathematical terms for the complex model

Symbol	Description	Vector
*n*	The number of links	
*l*	The half length of a link	
*m*	Mass of each link	
*J*	Moment of inertia of each link	
$\theta _{i}$	Angle between link *i* and the global *x* axis	${\varvec{\theta }}$ $\in \mathbb {R}^n$
$\phi _{i}$	Angle of joint *i*	${\varvec{\phi }}$ $\in \mathbb {R}^{n-1}$
$(x_i, y_i)$	Global coordinates of the CM of link *i*	$\mathbf {X},\mathbf {Y}$ $\in \mathbb {R}^n$
$(p_x, p_y)$	Global coordinates of the CM of the robot	$\mathbf {p}_\text {{CM}}$ $\in \mathbb {R}^2$
$u_{i}$	Actuator torque of joint between link *i* and link $i+1$	$\mathbf {u}$ $\in \mathbb {R}^{n-1}$
$u_{i-1}$	Actuator torque of joint between link *i* and link $i-1$	$\mathbf {u}$ $\in \mathbb {R}^{n-1}$
$f_{x,i}$	Fluid force on link *i* in *x* direction	${\mathbf {f_{x}}}$ $\in \mathbb {R}^n$
$f_{y,i}$	Fluid force on link *i* in *y* direction	${\mathbf {f_{y}}}$ $\in \mathbb {R}^n$
$\tau _i$	Fluid torque on link *i*	${\varvec{\tau }} \in \mathbb {R}^n$
$h_{x,i}$	Joint constraint force in *x* direction on link *i* from link $i+1$	${\mathbf {h_{x}}}$ $\in \mathbb {R}^{n-1}$
$h_{y,i}$	Joint constraint force in *y* direction on link *i* from link $i+1$	${\mathbf {h_{y}}}$ $\in \mathbb {R}^{n-1}$
$h_{x,i-1}$	Joint constraint force in *x* direction on link *i* from link $i-1$	${\mathbf {h_{x}}}$ $\in \mathbb {R}^{n-1}$
$h_{y,i-1}$	Joint constraint force in *y* direction on link *i* from link $i-1$	${\mathbf {h_{y}}}$ $\in \mathbb {R}^{n-1}$

### Kinematics of underwater snake robot

The snake robot is assumed to move in a virtual horizontal plane, fully immersed in water, and has *n*+2 degrees of freedom (*n* link angles and the *x*-*y* position of the robot). The *link angle* of each link $i \in \left\{ 1, \ldots , n\right\}$ of the snake robot is denoted by $\theta _i \in \mathbb {R}$, while the *joint angle* of joint $i \in \left\{ 1, \ldots , n-1\right\}$ is given by

2 $$\begin{aligned} \phi _i=\theta _i-\theta _{i-1}. \end{aligned}$$

The link angles and the joint angles are assembled in the vectors ${\varvec{\theta }}=\left[ \theta _1, \ldots , \theta _n \right] ^T \in \mathbb {R}^{n}$ and ${\varvec{\phi }}=\left[ \phi _1, \ldots , \phi _{n-1} \right] ^T \in \mathbb {R}^{n-1}$, respectively. The *heading* (or *orientation*) $\bar{\theta } \in \mathbb {R}$ of the snake is defined as the average of the link angles [6], i.e. as

3 $$\begin{aligned} \bar{\theta }=\frac{1}{n}\sum _{i=1}^{n}\theta _{i}. \end{aligned}$$

The global frame position $$\mathbf {p}_\text {{CM}}$$$$\in \mathbb {R}^2$$ of the CM (center of mass) of the robot is given by

4 $$\begin{aligned} {\mathbf {p}}_{\text {CM}}=\left[ \begin{array}{c} p_{x} \\ p_{y} \\ \end{array} \right] =\left[ \begin{array}{c} \frac{1}{nm}\sum _{i=1}^{n}mx_{i} \\ \frac{1}{nm}\sum _{i=1}^{n}my_{i} \\ \end{array} \right] =\frac{1}{n}\left[ \begin{array}{c} {\mathbf {e}}^T{\mathbf {X}} \\ {\mathbf {e}}^T{\mathbf {Y}} \\ \end{array} \right] . \end{aligned}$$

### Hydrodynamic modeling

As it has been noted in the bio-robotics community, underwater snake (eel-like) robots bring a promising prospective to improve the efficiency and maneuverability of modern-day underwater vehicles. However, the dynamic modeling of the contact forces is quite complicated compared to the modeling of the overall rigid motion for these robotic systems. Hence, the hydrodynamic modeling task presents a major challenge. A closed form solution was proposed in [2] to solve the hydrodynamic modeling problem using an analytical simplified form suited for the design of online control of underwater snake robots. The hydrodynamic modeling approach from [2] that is considered in this paper, takes into account both the linear and the nonlinear drag forces (resistive fluid forces), the added mass effect (reactive fluid forces), the fluid moments and current effects. In particular, in [2], it is shown that the fluid forces on all links can be expressed in vector form as

5 $$\begin{aligned} \begin{array}{ll} {\mathbf {f}}=\left[ \begin{array}{c} {\mathbf {f_{x}}}\\ {\mathbf {f_{y}}}\\ \end{array} \right] = \left[ \begin{array}{c} {\mathbf {f_{A_{x}}}}\\ {\mathbf {f_{A_{y}}}}\\ \end{array} \right] +\left[ \begin{array}{c} {\mathbf {f\,^{\text {I}}_{D_{x}}}}\\ {\mathbf {f\,^\text {I}_{D_{y}}}}\\ \end{array} \right] +\left[ \begin{array}{c} {\mathbf {f\,^\text {II}_{D_{x}}}}\\ {\mathbf {f\,^\text {II}_{D_{y}}}}\\ \end{array} \right] . \end{array} \end{aligned}$$

The vectors $\mathbf {f_{A_{x}}}$ and $\mathbf {f_{A_{y}}}$ represent the effects from added mass forces and are expressed as

6 $$\begin{aligned} \begin{array}{ll} \left[ \begin{array}{c} {\mathbf {f_{A_{x}}}}\\ {\mathbf {f_{A_{y}}}}\\ \end{array} \right] = &-\left[ \begin{array}{cc} \mu _n\left( \mathbf{S }_\theta \right) ^2 & -\mu _n\mathbf{S }_\theta \mathbf{C }_\theta \\ -\mu _n\mathbf{S }_\theta \mathbf{C }_\theta &{} \mu _n\left( \mathbf{C }_\theta \right) ^2 \\ \end{array} \right] \left[ \begin{array}{c} \ddot{\mathbf {X} }\\ \ddot{\mathbf {Y}}\\ \end{array} \right] \\ &{}-\left[ \begin{array}{cc} -\mu _n\mathbf{S }_\theta \mathbf{C }_\theta & -\mu _n\left( \mathbf{S }_\theta \right) ^2\\ \mu _n\left( \mathbf{C }_\theta \right) ^2 & \mu _n\mathbf{S }_\theta \mathbf{C }_\theta \\ \end{array} \right] \left[ \begin{array}{c} {\mathbf {V}}^a_x\\ {\mathbf {V}}^a_y\\ \end{array} \right] \dot{{\varvec{\theta }}},\\ \end{array} \end{aligned}$$

where $\mathbf {V}^a_x=\text {diag}\left( V_{x,1},\ldots ,V_{x,n}\right) \in \mathbb {R}^{n\times n}$, $\mathbf {V}^a_y=\text {diag}\left( V_{y,1},\ldots ,V_{y,n}\right) \in \mathbb {R}^{n\times n}$ and $[V_{x,i}, V_{y,i}]^T$ is the current velocity expressed in inertial frame coordinates. The vectors $\mathbf {f\,^{\text {I}}_{D_{x}}}$, $\mathbf {f\,^\text {I}_{D_{y}}}$ and $\mathbf {f\,^{\text {II}}_{D_{x}}}$, $\mathbf {f\,^\text {II}_{D_{y}}}$ represent the effects from the linear (7) and nonlinear drag forces (8), respectively, where the relative velocities are given by (9).

7 $$\begin{aligned} \begin{array}{ll} \left[ \begin{array}{c} {\mathbf {f\,^{\text {I}}_{D_{x}}}}\\ {\mathbf {f\,^\text {I}_{D_{y}}}}\ \end{array} \right] =-\left[ \begin{array}{cc} c_t\mathbf{C }_\theta & -c_n\mathbf{S }_\theta \\ c_t\mathbf{S }_\theta & c_n\mathbf{C }_\theta \\ \end{array} \right] \left[ \begin{array}{c} {\mathbf {V_{r_{x}}}} \\ {\mathbf {V_{r_{y}}}}\\ \end{array} \right] \end{array} \end{aligned}$$

8 $$\begin{aligned} \begin{array}{ll} \left[ \begin{array}{c} {\mathbf {f\,^\text {II}_{D_{x}}}}\\ {\mathbf {f\,^\text {II}_{D_{y}}}}\\ \end{array} \right] =-\left[ \begin{array}{cc} c_t\mathbf{C }_\theta & -c_n\mathbf{S }_\theta \\ c_t\mathbf{S }_\theta & c_n\mathbf{C }_\theta \\ \end{array} \right] \text {sgn}\left( \left[ \begin{array}{c} {\mathbf {V_{r_{x}}}}\\ {\mathbf {V_{r_{y}}}}\\ \end{array} \right] \right) \left[ \begin{array}{c} {\mathbf {V_{r_{x}}}}^2 \\ {\mathbf {V_{r_{y}}}}^2\\ \end{array} \right] \end{array} \end{aligned}$$

9 $$\begin{aligned} \left[ \begin{array}{c} {\mathbf {V_{r_{x}}}} \\ {\mathbf {V_{r_{y}}}}\\ \end{array} \right] = \left[ \begin{array}{cc} \mathbf{C }_\theta & \mathbf{S }_\theta \\ -\mathbf{S }_\theta & \mathbf{C }_\theta \\ \end{array} \right] \left[ \begin{array}{c} \dot{\mathbf {X} }-{\mathbf {V}}_x\\ \dot{\mathbf {Y}}-{\mathbf {V}}_y\\ \end{array} \right] \end{aligned}$$

In addition, the fluid torques on all links are

10 $$\begin{aligned} {\varvec{\tau }}=-{\varvec{\Lambda }}_1\ddot{{\varvec{\theta }}}-{\varvec{\Lambda }}_2\dot{{\varvec{\theta }}}-{\varvec{\Lambda }}_3\dot{{\varvec{\theta }}}|\dot{{\varvec{\theta }}}|, \end{aligned}$$

where ${\varvec{\Lambda }}_1=\lambda _1\mathbf {I}_n$, ${\varvec{\Lambda }}_2=\lambda _2\mathbf {I}_n$ and ${\varvec{\Lambda }}_3=\lambda _3\mathbf {I}_n$. The coefficients $c_t$, $c_n$, $\lambda _2$, $\lambda _3$ represent the drag forces parameters due to the pressure difference between the two sides of the body, and the parameters $\mu _n$, $\lambda _1$ represent the added mass of the fluid carried by the moving body. Note that the added mass parameter in the *x* direction is considered equal to zero ($\mu _t=0$), because the added mass of a slender body in the longitudinal direction can be neglected compared to the body mass [2].

### Equations of motion

This section presents the equations of motion for the underwater snake robot. In [43] it is shown that the acceleration of the CM may be expressed as

11 $$\begin{aligned} \begin{array}{ll} \left[ \begin{array}{c} \ddot{p}_{x}\\ \ddot{p}_{y}\\ \end{array} \right] &=-\mathbf {M}_p\left[ \begin{array}{cc} {\mathbf {k}}_{11}& \mathbf {k}_{12}\\ {\mathbf {k}}_{21} & \mathbf {k}_{22} \\ \end{array} \right] \left[ \begin{array}{c} l{\mathbf {K}}^T( \mathbf{C }_{\theta } \dot{{\varvec{\theta }}}^2+\mathbf{S }_{\theta } \ddot{{\varvec{\theta }}})\\ l{\mathbf {K}}^T( \mathbf S _{\theta } \dot{{\varvec{\theta }}}^2-\mathbf C _{\theta } \ddot{{\varvec{\theta }}}) \\ \end{array} \right] \\ &\quad -{\mathbf {M}}_p\left[ \begin{array}{cc} {\mathbf {k}}_{12}& -{\mathbf {k}}_{11}\\ {\mathbf {k}}_{22} & -{\mathbf {k}}_{21} \\ \end{array} \right] \left[ \begin{array}{c} {\mathbf {V}}_x^a\\ {\mathbf {V}}_y^a \\ \end{array} \right] \dot{{\varvec{\theta }}}+{\mathbf {M}}_p\left[ \begin{array}{c} {\mathbf {e}}^T{\mathbf {f_{Dx}}}\\ {\mathbf {e}}^T {\mathbf {f_{Dy}}}\\ \end{array} \right] , \end{array} \end{aligned}$$

where the detailed derivation of the matrix $\mathbf {M}_p$ and vectors $\mathbf {k}_{11}$, $\mathbf {k}_{12}$, $\mathbf {k}_{21}$ and $\mathbf {k}_{22}$ are given in [2, 43]. In addition, it is shown that under the influence of fluid forces (5) and torques (10), the equations of motion of the underwater snake robot are obtained by (11) and (12), with $\mathbf {f_{Dx}}=\mathbf {f\,^{\text {I}}_{D_{x}}}+\mathbf {f\,^{\text {II}}_{D_{x}}}$ and $\mathbf {f_{Dy}}=\mathbf {f\,^{\text {I}}_{D_{y}}}+\mathbf {f\,^{\text {II}}_{D_{y}}}$ representing the drag forces in *x* and *y* directions and $\mathbf {u} \in \mathbb {R}^{n-1}$ the control input. For more details and the derivation of the matrices $\mathbf {M}_{\theta }$, $\mathbf {W}_{\theta }$, $\mathbf {V}_{\theta }$, $\mathbf {K_{Dx}}$ and $\mathbf {K_{Dy}}$, see [43].

12 $$\begin{aligned} \mathbf {M}_{{\varvec{\theta }}}\ddot{{\varvec{\theta }}}+{\mathbf {W}}_{{\varvec{\theta }}}{{\dot{{\varvec{\theta }}}}}^2+{\mathbf {V}}_{\theta }\dot{{\varvec{\theta }}}+{\varvec{\Lambda }}_3|\dot{{\varvec{\theta }}}|\dot{{\varvec{\theta }}}+{\mathbf {K_{Dx}}}{\mathbf {f_{Dx}}}+{\mathbf {K_{Dy}}}{\mathbf {f_{Dy}}}={\mathbf {D}}^T{\mathbf {u}}, \end{aligned}$$

By introducing the state variable ${\mathbf {x}}=\left[ {{\varvec{\theta }}}^T, \,{\mathbf {p}}_{\text {CM}}^T, \, {{{\dot{{\varvec{\theta }}}}}}^T, \,{{\dot{\mathbf{p}}_{\text {CM}}}}^T\right] ^T \in \mathbb {R}^{2n+4}$, we can rewrite the model of the robot compactly in state space form as

13 $$\begin{aligned} \dot{\mathbf {x}}=\left[ \dot{{\varvec{\theta }}}^T, \,\dot{\mathbf {p}}_{\text {CM}}^T, \, {{{\ddot{{\varvec{\theta }}}}}}^T, \,{{\ddot{\mathbf {p}}_{\text {CM}}}}^T\right] ^T={\mathbf {F}}({\mathbf {x}},{\mathbf {u}}), \end{aligned}$$

where the elements of $\mathbf {F}(\mathbf {x},\mathbf {u})$ are found by solving (11) and (12) for $\ddot{\mathbf{p}}_{\text {CM}}$ and ${\ddot{{\varvec{\theta }}}}$, respectively.

#### *Remark 1*

It is interesting to note that if, in the dynamic model (11, 12), we set the fluid parameters to zero and replace the drag forces in *x* and *y* direction with ground friction models, then the model reduces exactly to the dynamic model of a land-based snake robot described in [6]. The underwater snake robot model is thus an extension of the land-based snake robot model, and may be used for amphibious snake robots moving both on land and in water.

## Control-oriented model of underwater snake robots

The fundamental properties of underwater snake robots that are investigated in this paper will be based on a control-oriented model of an underwater snake robot moving in a virtual horizontal plane that was developed in [44]. In particular, in [44] an extensive analysis of the complex model of underwater snake robots described in “A complex model of underwater snake robots” was performed and from this analysis a set of essential properties that characterize the overall motion of the underwater snake robot were derived. It was shown that the control-oriented modeling approach captures these essential properties, resulting in a less complex model that is well suited for control design and analysis purposes, and at the same time has the same essential properties as the complex model presented in “A complex model of underwater snake robots”. In this section we will give a brief description of this control-oriented model.

### Overview of the modeling approach

**Fig. 2:**
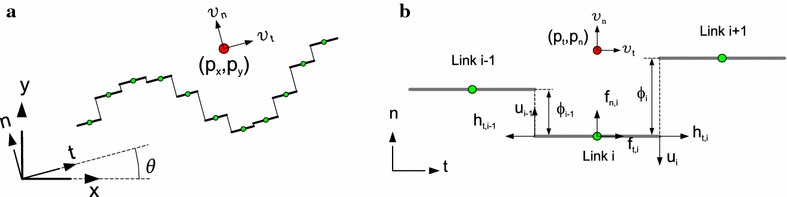
Parameters of the control-oriented model. **a** Control-oriented model approach and **b** kinematics and dynamics of the robot

The idea behind the control-oriented model of underwater snake robot locomotion is based on the simplified modeling approach presented in [6, 49] for a land-based snake robot. In particular, based on the observation that the rotation of each link in essence creates a linear displacement of the CM of each link, the idea is to describe the body shape changes of an underwater snake robot as linear displacements of the links with respect to each other instead of rotational displacements. The kinematics and dynamics of the underwater snake robot are given in terms of the mathematical symbols described in Table 2 and illustrated in Fig. 2. For further details, see [44]. Note that the control-oriented model is designed to capture only those properties of the underwater snake robot dynamics that are relevant for analysis and control design, and it is thus derived for control design and stability analysis purposes [44].

**Table 2:** Definition of mathematical terms for the control-oriented model

Symbol	Description	Vector
*N*	The number of links	
*l*	The length of a link	
*m*	Mass of each link	
$\phi _i$	Normal direction distance between links *i* and $i+1$	${\varvec{\phi }} \in \mathbb {R}^{N-1}$
$\upsilon _{\phi ,i}$	Relative velocity between links *i* and $i+1$	${\varvec{\upsilon }}_{\phi } \in \mathbb {R}^{N-1}$
$\theta$	Orientation of the underwater snake robot	$\theta \in \mathbb {R}$
$\upsilon _{\theta }$	Angular velocity of the underwater snake robot	$\upsilon _{\theta } \in \mathbb {R}$
$(t_i, n_i)$	Coordinates of the CM of link *i* in the $t-n$ frame	$(\mathbf {t}, \mathbf {n}) \in \mathbb {R}^{2N}$
$(p_t, p_n)$	Coordinates of the CM of the robot in the $t-n$ frame	$(p_t, p_n) \in \mathbb {R}^2$
$(p_x, p_y)$	Coordinates of the CM of the robot in the global frame	$(p_x, p_y) \in \mathbb {R}^2$
$(\upsilon _t, \upsilon _n)$	Forward and normal direction velocity of the robot	$(\upsilon _t, \upsilon _n) \in \mathbb {R}^2$
$u_{i}$	Actuator force at joint *i*	$\mathbf {u} \in \mathbb {R}^{N-1}$
$(f_{x,i}, f_{y,i})$	Fluid force on link *i* in the global frame	$(\mathbf {f_{x}}, \mathbf {f_{y}}) \in \mathbb {R}^{2N}$
$(f_{t,i}, f_{n,i})$	Fluid force on link *i* in the $t-n$ frame	$(\mathbf {f_{t}}, \mathbf {f_{n}}) \in \mathbb {R}^{2N}$

### Kinematics of the underwater snake robot

The underwater snake robot is assumed to move in a horizontal plane, fully immersed in water, and has *N*+2 degrees of freedom. The motion of the robot is defined with respect to the fixed global frame, $x-y$, and the $t-n$ frame that is always aligned with the robot (Fig.  2a). The origin of both frames are fixed and coincide. The direction of the *t* axis is denoted as the tangential or forward direction of the robot, and the direction of the *n* axis as the normal direction. As shown in Fig. 2, the position of the CM of the underwater snake robot in the global frame is denoted by $(p_x,p_y) \in \mathbb {R}^{2}$, while $(p_t,p_n) \in \mathbb {R}^{2}$ is the position in the $t-n$ frame. $\theta \in \mathbb {R}$ represents the orientation of the snake robot with respect to the global *x* axis with counterclockwise positive direction. The angle between the global *x* axis and the *t* axis is also $\theta$ since the $t-n$ frame is always aligned with the snake robot. The relationship between the $t-n$ frame position and the global frame position is thus given by

14 $$\begin{aligned} \begin{array}{ll} p_t&=p_x\cos \theta +p_y\sin \theta ,\\ p_n&=-p_x\sin \theta +p_y\cos \theta . \end{array} \end{aligned}$$

### Fluid dynamic model

The fluid dynamic model of the control-oriented model presented in [44] is notably less complex than the fluid dynamic model presented in “A complex model of underwater snake robots”, which takes into account significant parameters such as added mass effects, linear drag forces, torques due to the added mass and linear drag forces. In particular, in [44] it is shown that the expressions for the added mass effects and linear drag forces can be written as

15 $$\begin{aligned} \begin{array}{ll} \left[ \begin{array}{c} {\mathbf {f_{A_{t}}}}\\ {\mathbf {f_{A_{n}}}}\\ \end{array} \right] =&-\left[ \begin{array}{cc} {\mathbf {0}}_{N\times N} & -\dfrac{\mu _n}{2l} \text {diag}({\mathbf {A}}^T{\mathbf {\phi }}){\mathbf {e}}\\ -\dfrac{\mu _n}{2l} \text {diag}({\mathbf {A}}^T{\mathbf {\phi }}){\mathbf {e}} & \mu _n {\mathbf {I}}_N{\mathbf {e}} \\ \end{array} \right] \left[ \begin{array}{c} \dot{\upsilon }_t\\ \dot{\upsilon }_n\\ \end{array}\right] \\ &-\left[ \begin{array}{cc} {\mathbf {0}}_{N\times N} & -\dfrac{\mu _n}{2l} \text {diag}({\mathbf {A}}^T{\mathbf {\phi }})\\ -\dfrac{\mu _n}{2l} \text {diag}({\mathbf {A}}^T{\mathbf {\phi }}) & \mu _n {\mathbf {I}}_N \\ \end{array} \right] \left[ \begin{array}{c} {\mathbf {0}}_{N}\\ -\bar{\mathbf {D}}\ddot{\mathbf {\phi }}\\ \end{array}\right] \end{array} \end{aligned}$$

and

16 $$\begin{aligned} \left[ \begin{array}{c} {\mathbf {f_{D_{t}}}}\\ {\mathbf {f_{D_{n}}}}\\ \end{array} \right] =\left[ \begin{array}{c} -c_t\upsilon _t {\mathbf {e}}+c_p \text {diag}({\mathbf {A}}^T{\mathbf {\phi }})(\upsilon _n{\mathbf {e}}-\bar{\mathbf {D}}\dot{\mathbf {\phi }}) \\ -c_n\upsilon _n {\mathbf {e}}+c_n\bar{\mathbf {D}}\dot{\mathbf {\phi }}+c_p\upsilon _t\text {diag}({\mathbf {A}}^T{\mathbf {\phi }})\mathbf {e}\\ \end{array}\right] , \end{aligned}$$

respectively. The parameter $c_p={(c_n-c_t)}/2l$ is a propulsion coefficient which maps the normal direction link velocities and the joint coordinates into propulsive fluid forces in the forward (tangential) direction of the underwater snake robot, $I_N\in \mathbb {R}^{N \times N}$ is the unity matrix and $\mathbf {\bar{D}}=\mathbf {D}^T\left( \mathbf {D}\mathbf {D}^T\right) ^{-1}\,\in \mathbb {R}^{N\times N-1}$.

Furthermore, in [44] the fluid torques due to the added mass and linear drag effects are modeled as

17 $$\begin{aligned} \tau =-\tilde{\lambda }_1\dot{\theta }-\tilde{\lambda }_3\ddot{\theta }, \end{aligned}$$

where $\tilde{\lambda }_1$ is a constant parameter which determines the drag torque opposing to the rotation of the underwater snake robot and $\tilde{\lambda }_3$ is a constant parameter which represents the torque coefficient due to the added mass effect.

### Dynamics of the underwater snake robot

In [44], it is shown that by choosing the state vector of the model as

18 $$\begin{aligned} \mathbf {x}=\left[ \mathbf {\phi }^T, \theta , p_x, p_y, \mathbf {v}_{\phi }^T, \upsilon _{\theta }, \upsilon _t, \upsilon _n\right] ^T \quad \in \mathbb {R}^{2N+4}, \end{aligned}$$

the complete control-oriented model of the underwater snake robot is given by

19a $$\begin{aligned}&\dot{\mathbf {\phi }}=\mathbf {v}_{\phi } \end{aligned}$$

19b $$\begin{aligned}&\dot{\theta }=\upsilon _{\theta } \end{aligned}$$

19c $$\begin{aligned}&\dot{p}_x=\upsilon _t\cos \theta -\upsilon _n\sin \theta \end{aligned}$$

19d $$\begin{aligned}&\dot{p}_y=\upsilon _t\sin \theta +\upsilon _n\cos \theta \end{aligned}$$

19e $$\begin{aligned}&\dot{\mathbf {v}}_{\phi }=\left( -{c_nN}{\mathbf {v}}_{\phi } +{N} \left( {k_1}{\mathbf {A}}{\mathbf {D}}^T\dot{\upsilon }_t/{2}+c_p\mathbf {A}{\mathbf {D}}^T\upsilon _t \right) {\mathbf {\phi }} +{N}{\mathbf {D}}{\mathbf {D}}^T{\mathbf {u}}\right) /{k_{2}} \end{aligned}$$

19f $$\begin{aligned}&\dot{\upsilon }_{\theta }=-\dfrac{{\tilde{\lambda }_1}\upsilon _{\theta }}{1+\tilde{\lambda }_3}+\dfrac{{\tilde{\lambda }_2}\upsilon _t\bar{\mathbf {e}}^T{\mathbf {\phi }}}{(N-1)(1+\tilde{\lambda }_3)} \end{aligned}$$

19g $$\begin{aligned} \dot{\upsilon }_t&=k_3\left( k_12c_p(\bar{\mathbf {e}}^T{\mathbf {\phi }})^2-k_{2}c_tN\right) \upsilon _t+k_3\left( k_{2}2c_p\bar{\mathbf {e}}^T{\mathbf {\phi }}-k_1c_nN\bar{\mathbf {e}}^T{\mathbf {\phi }}\right) \upsilon _n\nonumber \\&\quad -k_3\left( k_{2}{k_1}{\mathbf {\phi }}^T{\mathbf {A}}\bar{\mathbf {D}}\dot{\mathbf {v}}_{\phi }/{2}+k_{2}c_p{\mathbf {\phi }}^T{\mathbf {A}}\bar{\mathbf {D}}{\mathbf {v}}_{\phi }\right) \end{aligned}$$

19h $$\begin{aligned} \dot{\upsilon }_n&=k_3\left( Nm2c_p\bar{\mathbf {e}}^T\mathbf {\phi }-k_1c_tN\bar{\mathbf {e}}^T\mathbf {\phi }\right) \upsilon _t+k_3\left( k_12c_p(\bar{\mathbf {e}}^T\mathbf {\phi })^2-N^2mc_n \right) \upsilon _n\nonumber \\&\quad -\bar{\mathbf {e}}^T\mathbf {\phi }k_3\left( k_1c_p{\mathbf {\phi }}^T{\mathbf {A}}\bar{\mathbf {D}}{\mathbf {v}}_{\phi }+{{k_1}^2}{\mathbf {\phi }}^T{\mathbf {A}}\bar{\mathbf {D}}\dot{\mathbf {v}}_{\phi }/{2}\right) \end{aligned}$$

where $\mathbf {\phi } \in \mathbb {R}^{N-1}$ are the joint coordinates, $\theta \in \mathbb {R}$ is the absolute orientation, $(p_x,p_y) \in \mathbb {R}^{2}$ is the position of the CM in the global frame and $\mathbf {v}_{\phi }=\dot{\mathbf {\phi }} \in \mathbb {R}^{N-1}$ are the joint velocities. The state variable $\upsilon _{\theta }=\dot{\theta } \in \mathbb {R}$ denotes the angular velocity, $(\upsilon _t, \upsilon _n) \in \mathbb {R}^{2}$ are the tangential and normal direction velocities of the robot, $\mathbf {u} \in \mathbb {R}^{N-1}$ are the transformed actuator forces and $$\mathbf {\bar{e}}=\left[ \begin{array}{ccc} 1&\ldots&1\end{array}\right] ^{T}\in \mathbb {R}^{N-1}$$. The parameters $k_1=\mu _n/l$, $k_2=Nm+N\mu _n$, $k_3=1/({Nmk_{2}-(k_1\bar{\mathbf {e}}^T\mathbf {\phi })^2})$ and $\tilde{\lambda }_2$ is a constant parameter which gives the scaling of the mapping from the average of the joint coordinates and forward velocity to rotational acceleration. For more detail, see [44]. The transformed actuator forces at the joints are chosen according to the feedback linearizing control law

20 $$\begin{aligned} \mathbf {u}=\dfrac{k_2}{N}\left( \mathbf {D}\mathbf {D}^T\right) ^{-1}\left( \bar{\mathbf {u}}+\dfrac{c_nN}{k_{2}}\dot{\mathbf {\phi }} - \dfrac{N}{k_{2}}\left( \dfrac{k_1}{2}\mathbf {A}\mathbf {D}^T\dot{\upsilon }_t+c_p\mathbf {A}\mathbf {D}^T\upsilon _t\right) \mathbf {\phi }\right) , \end{aligned}$$

where $\bar{\mathbf {u}} \in \mathbb {R}^{N-1}$ denotes the new control inputs. By using this control law the joint dynamics (19e) are transformed into

21 $$\dot{\mathbf {v}}_{\phi } = \bar{\mathbf {u}}$$

#### *Remark 2*

Note that in [44], it is shown that, for both lateral undulation and eel-like motion, the control-oriented model presented in this section and the complex model presented in “A complex model of underwater snake robots” have similar qualitative and quantitative behavior for $\theta _i<20{^{\circ }}$ (i.e., the modeling approach is limited to underwater snake locomotion where the link angles are limited) by choosing proper values for the fluid parameters of the control-oriented model. The similar behavior of the two models presented in [44] confirms that the control-oriented model can capture the significant effects that determine the overall motion of the underwater snake robot. Hence, the proposed control-oriented modeling approach can be used to develop a general analysis and control design, in order to get results that will also be applicable for the complex model.

## Joint controller

The fundamental properties that are investigated in this paper hold for underwater snake robots that follow a sinusoidal motion pattern. In this section we present a general sinusoidal motion pattern for underwater snake robots proposed in [45] and a control law for making the joint angles track the resulting joint reference angles.

### Motion pattern

The mathematical expression for the gait of the snake robot in locomotion studies depends on its construction and model. Previous studies on swimming snake robots have focused on two motion patterns; lateral undulation and eel-like motion. [45] proposes a general sinusoidal motion pattern, as a broader class of the aforementioned ones. Lateral undulation constitutes the fastest and most common type of ground snake locomotion [6, 50]. It is achieved by means of body waves, with a constant amplitude, propagated from head to tail, while the snake robot is commanded to follow the serpenoid curve [8]. Note that we include this motion pattern since it is commonly used for ground snake robots, and we therefore, want to show how this (forward) motion is obtained by an underwater snake robot. On the other hand, eel-like motion can be achieved by propagating lateral axial undulations with increasing amplitude from head to tail [51]. To achieve the general sinusoidal motion pattern, each joint $i \in \left\{ 1, \ldots , n-1\right\}$ of the underwater snake robot is commanded to track the reference signal

22 $$\begin{aligned} \phi _{i}^{*}(t)=\alpha \text {g}(i,n)\sin (\omega t+(i-1)\delta )+\phi _0, \end{aligned}$$

where $\alpha$ and $\omega$ are the maximum amplitude and the frequency, respectively, $\delta$ determines the phase shift between the joints, while the function $\text {g}(i,n)$ is a scaling function for the amplitude of joint *i*. This function allows (22) to describe a quite general class of sinusoidal functions, including several different snake motion patterns. For instance, $\text {g}(i,n)=1$ gives lateral undulation, while $\text {g}(i,n)=(n-i)/(n+1)$ gives eel-like motion [2]. Finally, the parameter $\phi _0$ is a joint offset coordinate that is met in land-based snake robots [6] and fish robots [52] as well, affecting the direction of locomotion in both cases.

### PD joint controller

In order to make the joint angle $\phi _i$ follow its reference signal $\phi _{i}^{*}$, a PD controller is used for both the complex and the control-oriented models:

23 $$\begin{aligned} u_{i}=\ddot{\phi }_{i}^{*}+k_{d}\left( {{\dot{\phi }}_{i}^{*}-\dot{\phi }}_{i}\right) +k_{p}\left( \phi _{i}^{*}-\phi _{i}\right) , \quad i \in \left\{ 1, \ldots , n-1\right\} \,, \end{aligned}$$

where $k_{p}>0$ and $k_{d}>0$ are the gains of the controller.

Note that for the experimental and the simulation results presented in the following sections the values of the gait parameters $\alpha$, $\omega$, $\delta$ in (22) and the controller gains, $k_p$, $k_d$ in (23) are chosen based on our experience with undulatory motion of underwater snake robots. In future work, optimization techniques may be used for choosing the optimal gait parameters and preferably the controller gains should be based on model-based analysis.

## Experimental setup

This section describes the experimental setup employed for the investigation of the relationship between the gait patterns, the forward velocity and the power consumption. Furthermore, the underwater snake robot that was used in our experiments is briefly presented. A more detailed description of the robot can be found in [24].

### Underwater snake robot—Mamba

**Fig. 3:**
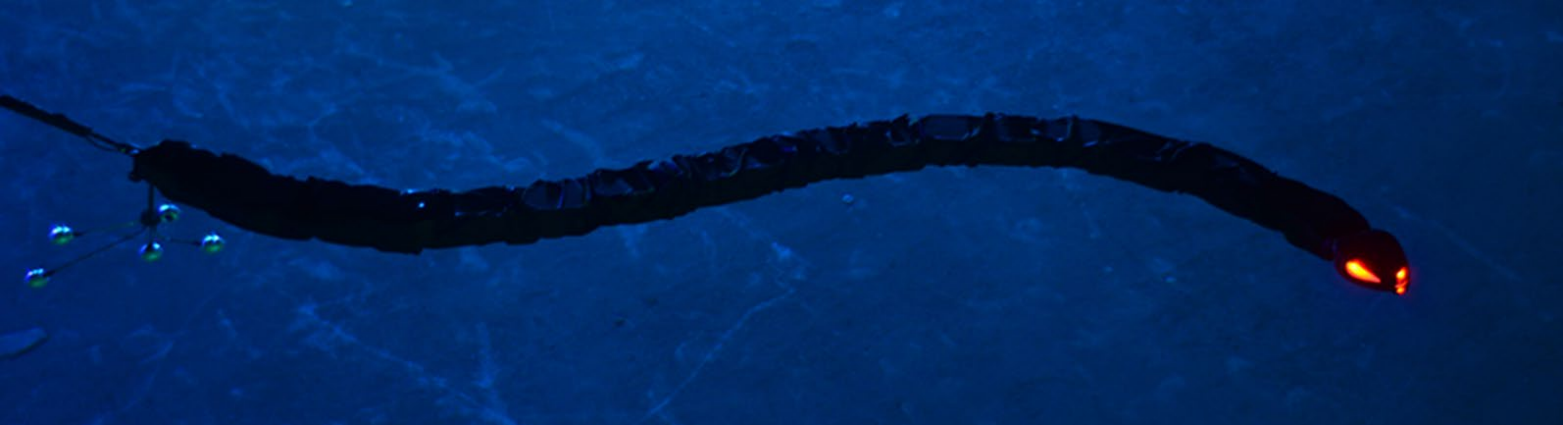
The underwater snake robot Mamba developed at NTNU to support the research on both ground and underwater snake robot locomotion. Markers are attached to the tail of the robot for position measurements

Mamba (Fig. 3) is a snake robot that is developed for research on both ground and underwater snake robot locomotion. This flexibility results from its mechanical robustness and reconfigurable nature. The robot is watertight and has a modular design with a common mechanical and electrical interface between the modules. Each joint module is actuated by a Hitec servo motor (HSR 5990TG) and also contains a force/torque sensor on the joint shaft, two temperature sensors, a 3-axis accelerometer, and a water leakage detector. Furthermore, each joint is controlled by means of a microcontroller card (TITechSH2 Tiny Controller from HiBot), and all microcontrollers in the robot communicate over a CAN bus. Power supply cables (35 V) run through all the modules along with the CAN bus.

Although the modules of the robot are watertight down to about 5 m, we covered the robot by a watertight skin during the experiments in order to achieve an additional water barrier (Fig. 3). The skin was custom designed from Groundsheet, Nylon, PU-coated, 120 g/m$^2$ material, and is sealed at the head and the tail parts using rubber bottle wrist seals, which are glued to the skin. This type of cover makes the robot’s outer surface more smooth, thus reducing the drag effects.

### Experimental setup

The experiments were performed in the MC-lab in Marintek, Trondheim, Norway [53], in a tank of dimensions *L*: 40 m, *H*: 1.5 m and *W*: 6.45 m. Real time measurements of the position and orientation of the robot was provided by an underwater motion capture system from Qualisys [54] installed in the basin. The system consists of six identical cameras, which allow reflective markers to be tracked under water inside a working area of dimensions 10 m $\times$ 1.35 m $\times$ 5.45 m.

The snake robot used in the experiments (see Fig. 3) consisted of 18 identical joint modules mounted horizontally and vertically in an alternating fashion [24]. In order for the robot to move according to a strictly horizontal motion pattern, the angles for the joints with vertical rotating axis were set to zero degrees. In this case, the kinematics of the snake robot corresponds to a planar snake robot with links of length $2l = 0.18$ m and mass $m \approx 0.8$ kg. The experiments demonstrated that the robot had a slightly positive buoyancy and was swimming near the water surface.

Tracking of the position and orientation of the robot by the motion capture system was achieved by mounting reflective markers on the tail part of the robot, as shown in Fig. 3. Although the robot was swimming near the water surface of the tank, the markers were submerged approximately 0.15 m under the water surface due to constraints in the work area covered by the camera system The global frame position and orientation of the tail link were measured in real time by the camera-based motion capture system. Having the measurements of the tail position and orientation, and the individual joint angles, the center of mass position, $\mathbf {p}_\text {CM}$, and the absolute link angles, ${\varvec{\theta }}$, of the robot were calculated from the kinematic equations presented in “A complex model of underwater snake robots”.

## Relationships between gait parameters, the forward velocity and the power consumption

The fundamental properties of underwater snake robots that will be investigated numerically and experimentally in this paper were developed in [45] and [46]. In particular, the relationship between the gait parameters in the general sinusoidal motion pattern given by (22) and the forward velocity was found in [45], and the relationship between the gait parameters and the average power consumption was found in [46]. In “Simulation study: relationships between gait parameters and forward velocity” and “Experimental study: relationships between gait parameters and forward velocity”, simulation and experimental results will be presented to investigate the validity of these properties for the underwater snake robot locomotion. In this section we present the fundamental properties, while details about their derivation can be found in [45, 46].

### Fundamental properties derived based on averaging theory

The joint motion of swimming biological snakes is periodic, and snake robots adapt the same motion pattern (see [2]). The control-oriented model presented in “Control-oriented model of underwater snake robots” is specifically designed to capture this motion through capturing the corresponding translational motion during oscillations. Based on this control-oriented model, in [45], averaging theory was applied to derive the average velocity dynamics of the underwater snake robot in the general case when it moves according to the sinusoidal motion patterns described by (22). The results in [45] are based on the hypothesis that oscillatory behavior causes some averaged effect that forces the robot to move forward. The averaging theory is extensively used for analysis of locomotion of biomimetic systems with oscillatory inputs, and it is applied in several works to study the locomotion of snake or fish robots [6, 27, 34, 55–57].

In [45], the averaged model of the velocity dynamics was found, and based on this the stability properties for general sinusoidal motion gait patterns were investigated. In particular, it was shown that the average velocity of an underwater snake robot following a sinusoidal motion pattern converges exponentially to a steady state velocity. An analytical expression for calculating this steady state velocity was presented as a function of the gait pattern parameters. In particular, it was shown that the resulting steady state velocity of the underwater snake robot in addition to depending on the parameters of the robot (i.e. *m*, *l*, *N*, $\mu _n$, $c_n$, $c_t$, $\tilde{\lambda }_1$, $\tilde{\lambda }_2$, $\tilde{\lambda }_3$), also depend on the sinusoidal gait pattern parameters $\alpha$, $\omega$, $\delta$ and $\phi _0$.

The results presented in [45] are summarized in the following proposition.

#### **Proposition 1**

*Consider an underwater snake robot withN links described by* (19),* influenced both by added mass and linear drag effects, that follows any sinusoidal gait pattern described by * (22).* The average forward velocity of the underwater snake robot will converge exponentially to a steady state velocity which:*

1. *Is a function of the amplitude of the sinusoidal motion pattern, *$\alpha$.
2. *Depends on a linear and a nonlinear function of the gait frequency,*$\omega$.
3. *Depends on the phase shift between the joints,*$\delta$.

#### *Remark 3*

Note that similar studies are presented for the special case of lateral undulation motion pattern for land-based snake robots [6] and for eel-like robots [34]. In particular, earlier studies for land-based snake robots [6] show that the average forward velocity of the robot is: (1) proportional to the square of the amplitude of the sinusoidal motion pattern, (2) proportional to the gait frequency and (3) depends also on the weighted sum of the constant phase shift between the joints. Moreover, similar study for a 5 link eel-like robot, where the added mass effects and fluid torques are neglected [34], predict that the acceleration should vary: (1) quadratically with gait amplitude, (2) linearly with gait frequency, and (3) accordingly to a weighted sum of sinusoids of the phase shift. However, Proposition 1 states that the average forward velocity of an underwater snake robot, influenced both by added mass and linear drag effects, and following a more general sinusoidal gait pattern, has a more complex relationships to the gait pattern parameters $\alpha$, $\omega$ and $\delta$. Therefore, the analytical study presented in [45], extend the previous studies presented for land-based snake robot and eel-like robot. In particular, the properties for a land-based snake robot presented in [6] fall out as a special case for the lateral undulation motion pattern, where the fluid parameters due to the added mass are set to zero and when an anisotropic ground friction model is considered instead of a drag friction model. Moreover, the results presented in [34] can be considered as a special case compared to the results presented in Proposition 1 by neglecting the added mass effects and considering a swimming robot with 5 links.

#### *Remark 4*

The derived relationship between the gait pattern parameters and the steady state velocity presented in Proposition 1 provides a useful tool for motion planning and parameter tuning of sinusoidal gait patterns for underwater snake robots. This information is useful since an increase/decrease of the forward velocity can be achieved by increasing/decreasing the gait parameters. The results presented in Proposition 1 are general and constitute a powerful tool for achieving faster forward motion by selecting the most appropriate motion pattern and the best combination of the gait parameters.

### Empirical rules derived based on simulation studies

Based on the closed form model which is briefly presented in “A complex model of underwater snake robots”, empirical rules are derived in [46] through an extensive simulation study for the relationships between the parameters of the gait patterns, the consumed energy and the forward velocity for different motion patterns for underwater snake robots. In particular [46], presents preliminary results by investigating the power consumption of different motion patterns for underwater snake robots. Based on the simulation results, empirical rules for choosing the values for the parameters of the motion gait pattern of underwater snake robots were proposed. The purpose of this study was to investigate the issues that influence the performance of underwater snake robots, both when it comes to the achieved forward velocity (moving performance) and the energy efficiency (transportation performance). In particular, the energy index [58] was used to compare the energy efficiency of underwater snake robots for different motion patterns. A similar approach is used to indicate the relationship between the mechanical index and the energy index of different transformation modes for ships in [58]. Comparison results were obtained for the average power consumption and the cost of transportation of underwater snake robots for different motion patterns.

Propositions 2 and 3 summarize the empirical rules proposed in [46], and which will be investigated through simulation and experimental studies in “Simulation study: relationships between gait parameters and forward velocity” and “Experimental study: relationships between gait parameters and forward velocity”. These rules can be used to choose the parameters of the gait patterns for underwater snake robots to achieve energy efficient motion while reaching the fastest possible forward velocity.

#### **Proposition 2**

*Given an underwater snake robot with n links described by* (11, 12)* which is controlled by* (23)* with the joint reference angles given by* (22).* The following rules hold for the forward velocity:*

1. *The forward velocity increases by increasing the parameter*$\omega$,* when *$\alpha$* is kept constant.*
2. *The forward velocity increases by increasing the parameter*$\alpha$,* when*$\omega$* is kept constant.*
3. *The forward velocity increases by increasing the parameter*$\omega$* and by increasing the amplitude*$\alpha$,* when*$\delta$* is kept constant.*
4. *There exists a value of the phase shift,*$\delta _{max}$,* that gives the maximum forward velocity. The forward velocity increases with increasing*$\delta$* for *$\delta < \delta _{max}$,* and decreases with *$\delta$* when*$\delta > \delta _{max}$,* and the maximum forward velocity is achieved when*$\delta = \delta _{max}$,* when*$\alpha$* and *$\omega$* are kept constant.*

#### **Proposition 3**

*Given an underwater snake robot withn links described by* (11, 12)* which is controlled by* (23)* with the joint reference angles given by* (22).* The following rules hold for the average power consumption:*

1. *The average power consumption decreases by increasing the parameter*$\delta$* when *$\alpha$* and*$\omega$* are kept constant, and it increases by increasing the parameter *$\omega$* when*$\alpha$* and *$\delta$* are kept constant.*
2. *The average power consumption decreases by increasing the parameter *$\delta$* when*$\alpha$* and*$\omega$* are kept constant and increases by increasing the parameter*$\alpha$* when *$\omega$* and*$\delta$* are kept constant.*
3. *The average power consumption increases by increasing the parameter*$\omega$* and by increasing the amplitude*$\alpha$,* when*$\delta$* is kept constant.*

In previous sections, we gave an integrated description of the modeling and the theoretical findings in the preliminary conference papers [2, 43–47] into a unified presentation in order to create the foundation which will now be used as basis for an experimental validation study in the following sections. In particular, simulation and experimental results will be presented in following sections to investigate the validity of the fundamental properties for underwater snake robot locomotion presented in Propositions 1–3.

## Simulation study: relationships between gait parameters and forward velocity

In this section, the validity of Propositions 1 and 2 will be investigated through a simulation study. In particular, we will present simulation results in order to validate the properties derived for the relationship between the gait pattern parameters and the forward velocity both for lateral undulation and eel-like motion patterns. Simulation results will be presented both for the control-oriented model and the complex model.

The simulation study will thus investigate the validity of the theoretical results in Propositions 1 and 2, and in addition the study will further investigate the validity of the control-oriented model as an adequate representation of the dynamics of the complex model, by investigating whether the results developed based on the control-oriented model also hold for the original complex model. In this study, current effects have not been considered, since current effects are not taken into account in the control-oriented model. The models were implemented in *Matlab R2013b*. The dynamics was calculated using the *ode23tb* solver with a relative and absolute error tolerance of $10^{-4}$.

### Simulation parameters

We consider snake robots with, respectively, $N = 5$, $N = 10$, $N = 20$ links, each one having length $l = 0.14$ m. The five links constitute a rather short snake robot, while ten to twenty links constitute a more normal length of snake robots. The mass of each link is $m = 0.6597$ kg and is chosen so to fulfil the neutrally buoyant assumption. The initial values of the states of the snake robot were set to the initial reference values at $t=0$, since it is not the transient behavior of the controller (20) that is to be verified, but rather the relationship between the gait pattern parameters and the forward velocity when the joints follow the sinusoidal reference signal (22). The initial heading of the robot is along the inertial *x* axis. Furthermore, we choose the fluid force and torque coefficients as $c_t=0.2639$, $c_n=4.2$, $\mu _n=0.3957$, $\lambda _1=2.2988\times 10^{-7}$, $\lambda _2=4.3103\times 10^{-4}$, for the complex model and $c_t=0.45$, $c_n=5$, $\mu _n=0.4$, $\tilde{\lambda }_1=0.5$, $\tilde{\lambda }_2=20$, $\tilde{\lambda }_3=0.01$ for the control-oriented model. An extensive discussion about the values of the fluid parameters can be found in [45]. The joint PD controller (23) is used for each joint with parameters $k_{p}=20$, $k_{d}=5$, and lateral undulation and eel-like motion are achieved by choosing $\text {g}(i,n)=1$ and $\text {g}(i,n)=(n-i)/(n+1)$, respectively. The gait pattern parameters are presented in each simulation result. In particular, in the simulation results the forward velocity of the underwater snake robot, denoted as $\bar{\upsilon }$, is presented for different values of the gait parameters. The forward velocity can be calculated based on the initial and final position. In particular, for each simulation trial with simulation time set to 30 s the average forward velocity is given by

24 $$\begin{aligned} \bar{\upsilon }=\dfrac{\sqrt{(p_x(30)-p_x(0))^2+(p_y(30)-p_y(0))^2}}{30} \end{aligned}$$

#### *Remark 5*

Even though the joint coordinates of the control-oriented model (linear translations) and the complex model (joint angles) are described using different physical quantities, it still makes sense to investigate the validity of Proposition 1 for the complex model (see [6]).

#### *Remark 6*

Note that the values of the gait parameter $\alpha$ for the complex model does not correspond directly to $\alpha$ for the control-oriented model, since a general mapping for the amplitudes of the corresponding models remains a topic for future work, and thus a quantitative comparison between the results from the complex and the control-oriented model is not relevant. Hence, the simulation results below present only a qualitative comparison between the complex and the control-oriented models.

### Relationship between $\alpha$ and the forward velocity

**Fig. 4:**
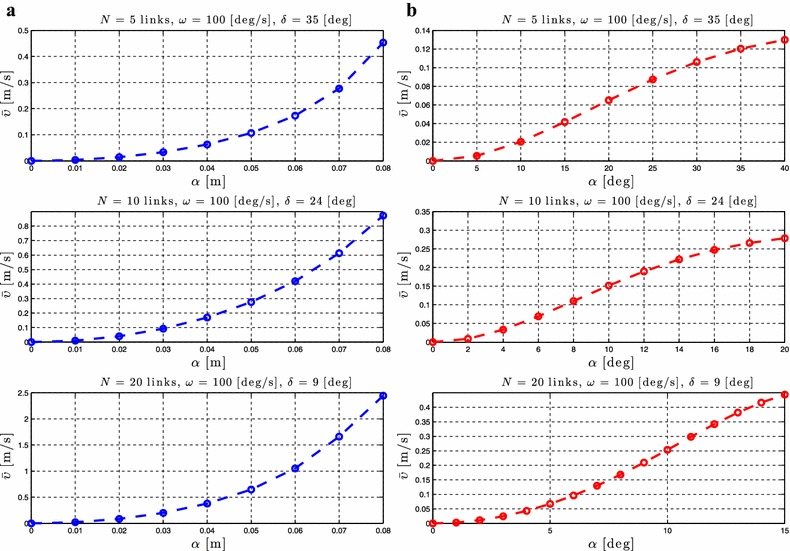
Lateral undulation: simulation results for the forward velocity of the underwater snake robot for *different values* of $\alpha$. **a** Control-oriented model and **b** complex model

**Fig. 5:**
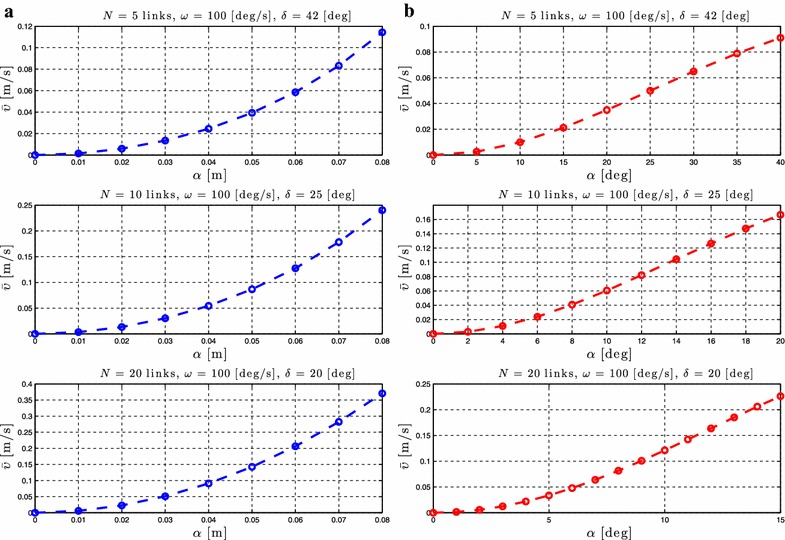
Eel-like motion: Simulation results for the forward velocity of the underwater snake robot for *different values* of $\alpha$. **a** Control-oriented model and **b** complex model

As stated in Proposition 1, the average forward velocity is a function of the amplitude of the sinusoidal motion pattern, $\alpha$. In order to investigate the influence of this parameter to the achieved forward velocity of the underwater snake robot, simulation results are presented for different values of the parameter $\alpha$ for both lateral undulation and eel-like motion patterns. The average forward velocity is calculated according to (24). Simulation results for the control-oriented model taking into account the added mass and linear drag effects are presented in Figs. 4a and 5a for lateral undulation and eel-like motion patters, respectively. Figures 4b and 5b presents simulation results for the complex model of underwater snake robot where the added mass, linear and nonlinear drag effects are taken into account, respectively, for lateral undulation and eel-like motion patterns. The number of links *N* and the values of the gait parameters $\omega$ and $\delta$ are shown in each simulation result for the different motion patterns. Note that we have different ranges of values for $\alpha$ for the complex model in Figs. 4b and 5b. This is because we need to decrease the amplitude of the angles when we increase the number of links to avoid collision between the links.

From Figs. 4 and 5, we see that the average forward velocity is increased by increasing the parameter $\alpha$ for constant values of $\omega$ and $\delta$ both for lateral undulation and eel-like motion. Note that for small values of the parameter $\alpha \le 20{^{\circ }}$, for which the control-oriented model is valid (see Remark 2), the forward velocity has an increase when increasing the parameter $\alpha$. Furthermore, we can see that even if the properties in Proposition 1 are derived based on the control-oriented model of underwater snake robots, the results for the complex model show a similar influence of the parameter $\alpha$ on the forward velocity. In particular, we see that for constant values of $\omega$ and $\delta$ an increase of the parameter $\alpha$ results in an increase of the forward velocity for both the complex and the control-oriented models. These results are in accordance with the properties presented in Propositions 1 and 2.

### Relationship between $\omega$ and the forward velocity

**Fig. 6:**
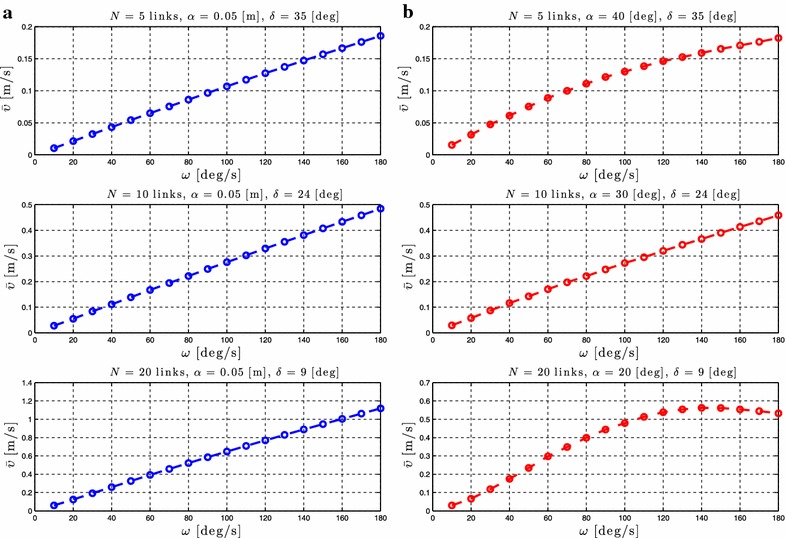
Lateral undulation: simulation results for the forward velocity of the underwater snake robot for *different values* of $\omega$. **a** Control-oriented model and **b** complex model

**Fig. 7:**
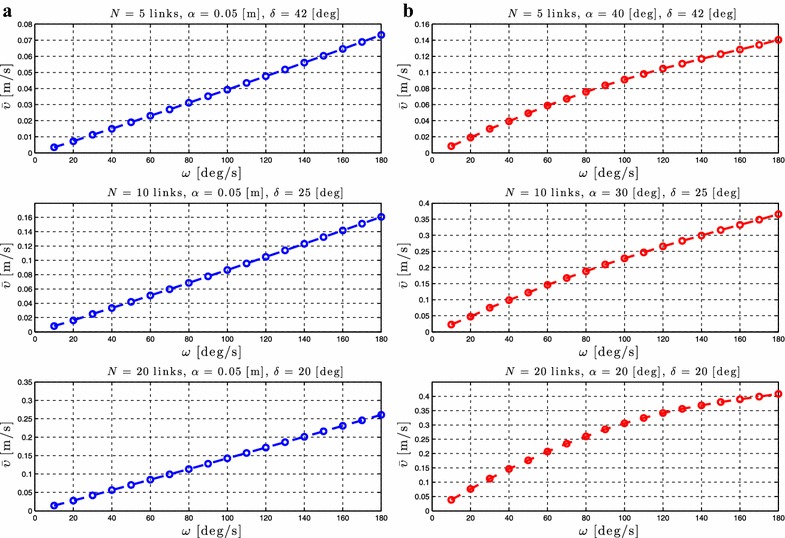
Eel-like motion: Simulation results for the forward velocity of the underwater snake robot for *different values* of $\omega$. **a** Control-oriented model and **b** complex model

Proposition 1 states that the average forward velocity depends on a linear and a nonlinear function of the gait frequency, $\omega$. To validate the influence of this parameter, simulation results are presented by calculating the forward velocity of the robot for different values of the gait parameter $\omega$. Simulation results for the control-oriented model are shown in Figs. 6a and 7a, while Figs. 6b and 7b show simulation results for the complex model presented in “A complex model of underwater snake robots”. The number of links *N* and the values of the gait parameters $\alpha$ and $\delta$ are shown in each simulation result for the different motion patterns.

From Figs. 6 and 7, we can see that for constant values of the parameters $\alpha$ and $\delta$ an increase of $\omega$ results in an increase of the forward velocity. This is in accordance with Proposition 1 which states that the average forward velocity depends on a linear and a nonlinear function of the parameter omega. In addition, from Figs. 6a and 7a, we see that the increase of the forward velocity is almost linear for both lateral undulation and eel-like motion patterns. Hence, the influence of the nonlinear function of the parameter $\omega$ on the forward velocity is almost negligible compared to the linear relationship between these given in Proposition 1 for the control-oriented model. However, in Figs. 6b and 7b, we clearly see the influence of the effects of a nonlinear function of $\omega$. Except for the high frequency case there is a good qualitative agreement between the simulation results of the complex and the control-oriented model. This is probably because the nonlinear drag effects that are considered in the complex model are not taken into account in the control-oriented model, and the nonlinear drag seems to have a dominating effect at high frequencies. The simulation results show that the properties derived based on the control-oriented model hold also for the complex model presented in “A complex model of underwater snake robots”, something which supports the assumption that the control-oriented model is a valid approximation of the complex model for analysis and control design. These results are in accordance with Proposition 1, which states that the average forward velocity is function of a linear and nonlinear terms of the parameter $\omega$.

### Relationship between $\delta$ and the forward velocity

**Fig. 8:**
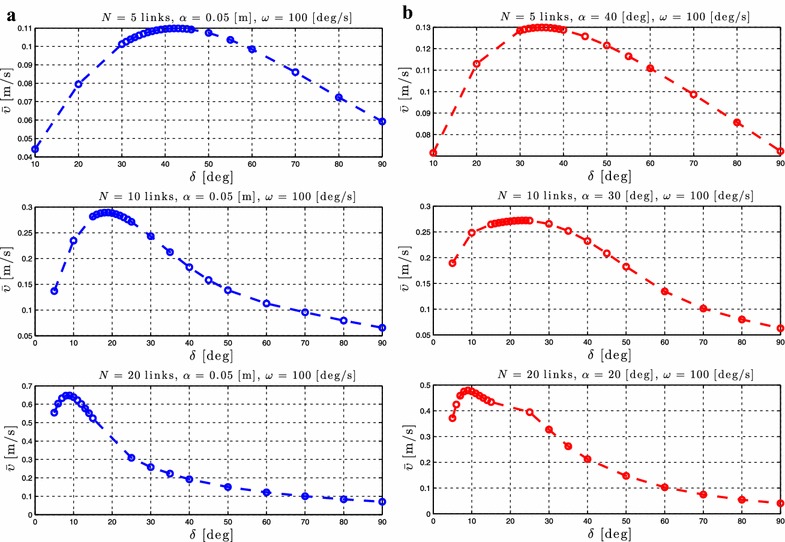
Lateral undulation: simulation results for the forward velocity of the underwater snake robot for *different values* of $\delta$. **a** Control-oriented model and **b** complex model

**Fig. 9:**
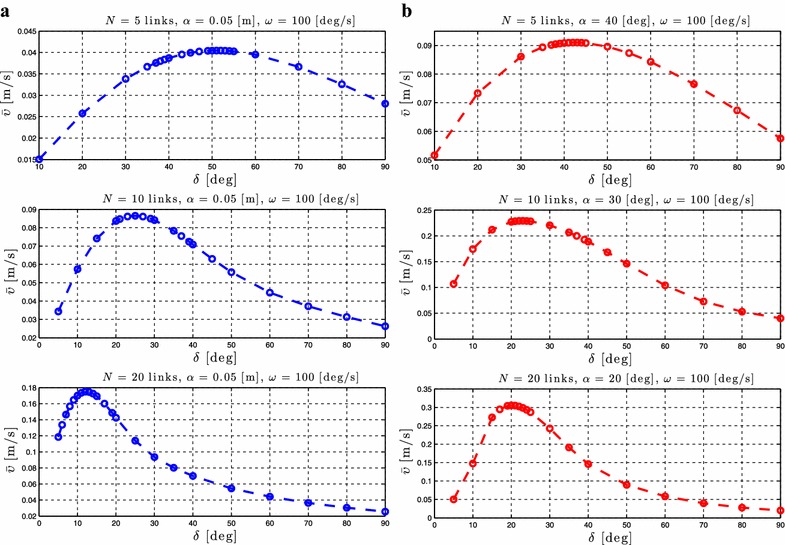
Eel-like motion: Simulation results for the forward velocity of the underwater snake robot for *different values* of $\delta$. **a** Control-oriented model and **b** complex model

Regarding the influence of the parameter $\delta$, Proposition 1 states that the forward velocity depends on the phase shift between the joints, $\delta$. To investigate the influence of the phase shift on the achieved forward velocity, simulation results are presented for different values of $\delta$ while keeping the gait parameters $\alpha$ and $\omega$ constant. Simulation results for the control-oriented model and the complex model are presented in Figs. 8a, b and 9a, b, respectively, for lateral undulation and eel-like motion patterns. The number of links *N* and the values of the gait parameters $\alpha$ and $\omega$ are shown in each simulation result for the different motion patterns.

From Figs. 8, 9, we see that there is a value of the gait parameter $\delta$ which gives the maximum forward velocity when the gait parameters $\alpha$ and $\omega$ are kept constant. This is in accordance with Proposition 2. In addition, we see that the forward velocity depends of the values of the parameter $\delta$, which is in accordance with the properties presented in Proposition 1. In particular, we see that we have an increase of the forward velocity until a certain value of the parameter $\delta$, while after this value an additional increase of this parameter for constant values of $\alpha$ and $\omega$ results in a decrease in the forward velocity. Note that the results presented in Figs. 8 and 9 for the complex and the control-oriented models are only qualitatively comparable as pointed out in Remark 5. However, the qualitative comparison supports that the control-oriented model is an adequate representation of the complex model, i.e. that the properties stated in Propositions 1 and 2 hold for both the control-oriented and the complex model.

Note that the analytical equation for the forward velocity that was derived in [45] based on the control-oriented model is a complex function of many parameters which involve the snake robot characteristics (i.e. *N*, *m*, *l*), the fluid parameters (i.e. $c_t$, $c_n$, $\mu _n$, $\tilde{\lambda }_1$, $\tilde{\lambda }_2$, $\tilde{\lambda }_3$) and the gait pattern parameters (i.e. $\alpha$, $\omega$, $\delta$, $\phi _0$). Due to this complexity it was not possible to obtain an analytical study that could provide more precise information regarding the relationship between the forward velocity and the parameters $\alpha$ and $\omega$ than the ones presented in Proposition 1. In particular, the preliminary results investigated in [45], and which were summarized in Proposition 1, stated that the forward velocity is a function of the parameter $\alpha$ and depends on a linear and a nonlinear term of the gait parameter $\omega$, but properties of these functions were not possible to derive analytically. However, based on the extensive simulation studies presented in this paper for underwater snake robot with different characteristics and for a wide range of the fluid parameters it is possibly to see the characteristics of these functions and thus to describe more precisely how the different parameters affect the achieved forward velocity. In particular, we employed simulation results for robots with different number of links, mass, and length of links, for different values of fluid parameters, varying the gait parameters and based on these simulation results we are able to show the actual dependence of the forward velocity on $\alpha$ and $\omega$. Figures 4a, 5a show that the forward velocity increases when the gait parameter $\alpha$ increases. Since the function in Proposition 1 (1) also is continuous in $\alpha$, the function is a class $\mathcal {K}$ function. From Figs. 6a, 7a we can see that the the forward velocity increases linearly when increasing the gait parameter $\omega$. Moreover, Figs. 6a, 7a clearly show that the linear term of $\omega$ dominates and the influence of the nonlinear term of $\omega$ is negligible.

The following proposition summarizes the above discussion:

#### **Proposition 4**

*Consider an underwater snake robot withN links described by* (19),* influenced both by added mass and linear drag effects, that follows any sinusoidal gait pattern described by* (22).* The average forward velocity of the underwater snake robot will converge exponentially to a steady state velocity which:*

1. *Is a class*$\mathcal {K}$* function of alpha, i.e., increases when the amplitude of the gait pattern *$\alpha$* increases, for small amplitudes*.
2. *Increases almost linearly with respect to the frequency of the gait pattern*$\omega$* (i.e., the nonlinear term of *$\omega$* has a negligible effect on the achieved forward velocity).*
3. *Depends on the phase shift between the joints*$\delta$.

#### *Remark 7*

The properties in Proposition 4 presented in this paper extend the ones stated in Proposition 1. In particular, a more accurate and precise relationship between the gait parameters and the achieved forward velocity is obtained and introduced in Proposition 4.

## Experimental study: relationships between gait parameters and forward velocity

In this section, experimental results will be presented to investigate the validity of the properties presented in Propositions 1, 4 and 2. In particular, we will experimentally validate the properties regarding the gait parameters derived in [45] and the empirical rules proposed in [46] using the underwater snake robot Mamba (Fig. 3). The underwater snake robot Mamba and the experimental setup were presented in “Experimental setup”.

### Simulation results

In order to compare the experimental results with ideal simulation results, we simulate the model of the underwater snake robot presented in “A complex model of underwater snake robots” with the fluid coefficients set to $$C_f=0.03$$, $$C_D=1$$, $$C_A=1$$, $$C_M=1$$ to compare the experimental results and the ideal simulation results. We consider an underwater snake robot with $$n =9$$ links, each one having length $$2l = 0.18$$ m and mass $$m = 0.8$$ kg, i.e. identical to the physical robot presented in “Experimental setup”. The hydrodynamic related parameters $$c_t$$, $$c_n$$, $$\mu _n$$$$\lambda _1$$, $$\lambda _2$$ and $$\lambda _3$$ for the elliptic section with major and minor diameters $$2a= 2\cdot 0.055$$ m and $$2b= 2\cdot 0.05$$ m, respectively, and $$\rho =1000$$ kg/m$$^3$$ were calculated by using equations derived in [2]. In these simulations, a joint PD-controller (23) was used with parameters $$k_{p}=20$$, $$k_{d}=5$$, while lateral undulation or eel-like motion were achieved by moving the joints according to (22) by choosing $$\text {g}(i,n)=1$$ and $$\text {g}(i,n)=(n-i)/(n+1)$$, respectively, with gait parameter values similar to the ones of the experimental trials.

#### *Remark 8*

Please note that accurate experimentally identified fluid parameters are not available for the physical robot presented in “Experimental setup”, and therefore the hydrodynamic parameters in the simulations differ from those of the experiments. To this end, we can only achieve a qualitative comparison and not a quantitative comparison between the simulation and experimental results.

### Experimental results

The essential properties presented in “Relationships between gait parameters, the forward velocity and the power consumption” were experimentally investigated using the underwater snake robot Mamba (see Fig. 3). As mentioned in “Underwater snake robot—Mamba” the robot consist of 18 identical joint modules mounted horizontally and vertically in an alternating fashion. The center of mass position, $\mathbf {p}_\text {CM}$, and the absolute link angles, ${\varvec{\theta }}$, of the underwater snake robot were obtained as described in “Experimental setup”. We applied sinusoidal motion patterns with different gait pattern parameters. In particular, in each trial, the reference joint angles, computed by (22) for $n=9$ choosing $\text {g}(i,n)=1$ and $\text {g}(i,n)=(n-i)/(n+1)$ for lateral undulation or eel-like motion, respectively, were sent to the robot via the CAN. In each trial we measured the position of the center of mass and the steady state values of the achieved velocity for approximately 30 sec of motion. A proportional controller, implemented in the microcontroller of each joint module controls the corresponding joint angle.

**Fig. 10:**
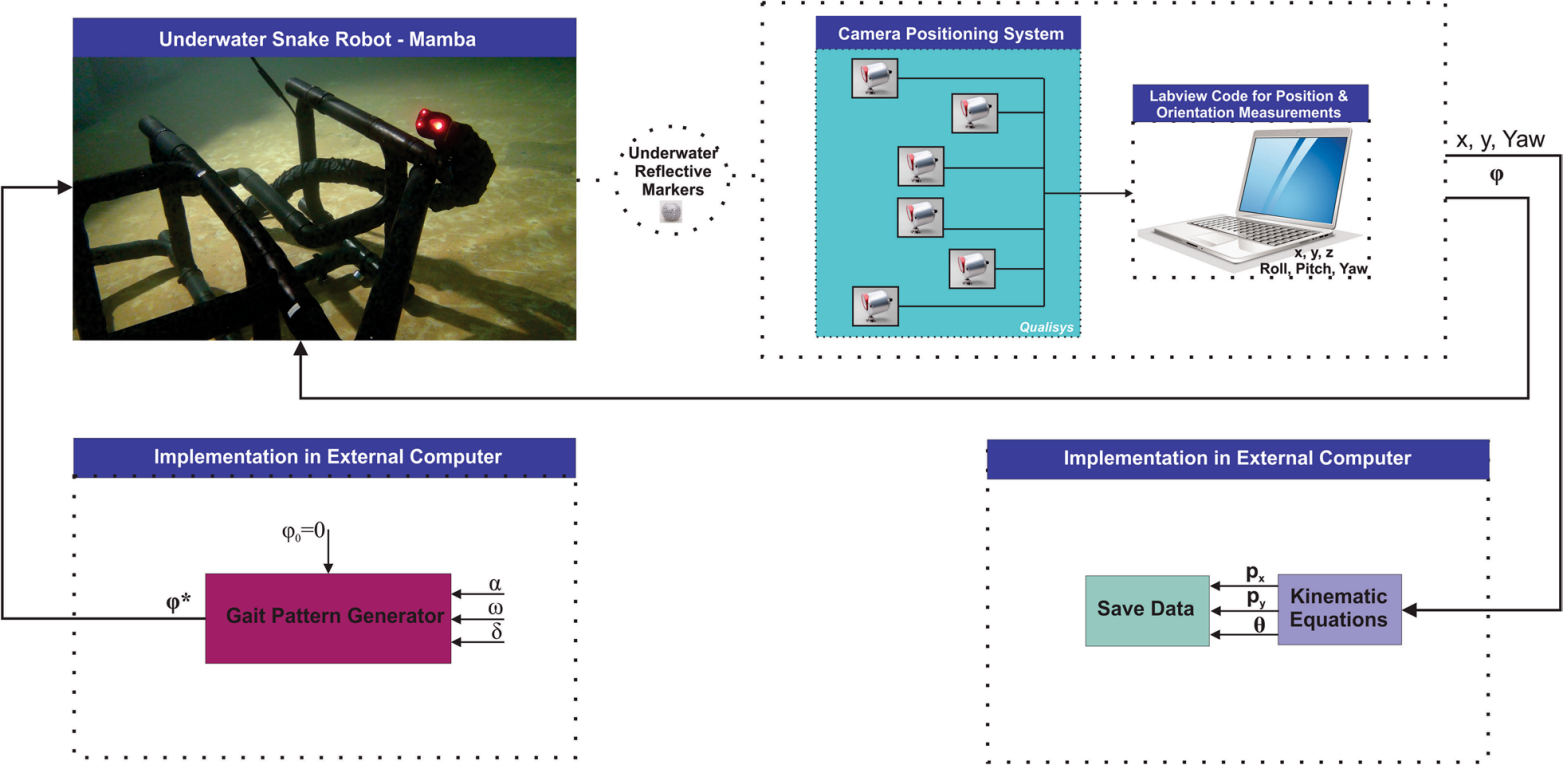
Illustration of the experimental process adopted in the experiments with the underwater snake robot Mamba

**Fig. 11:**
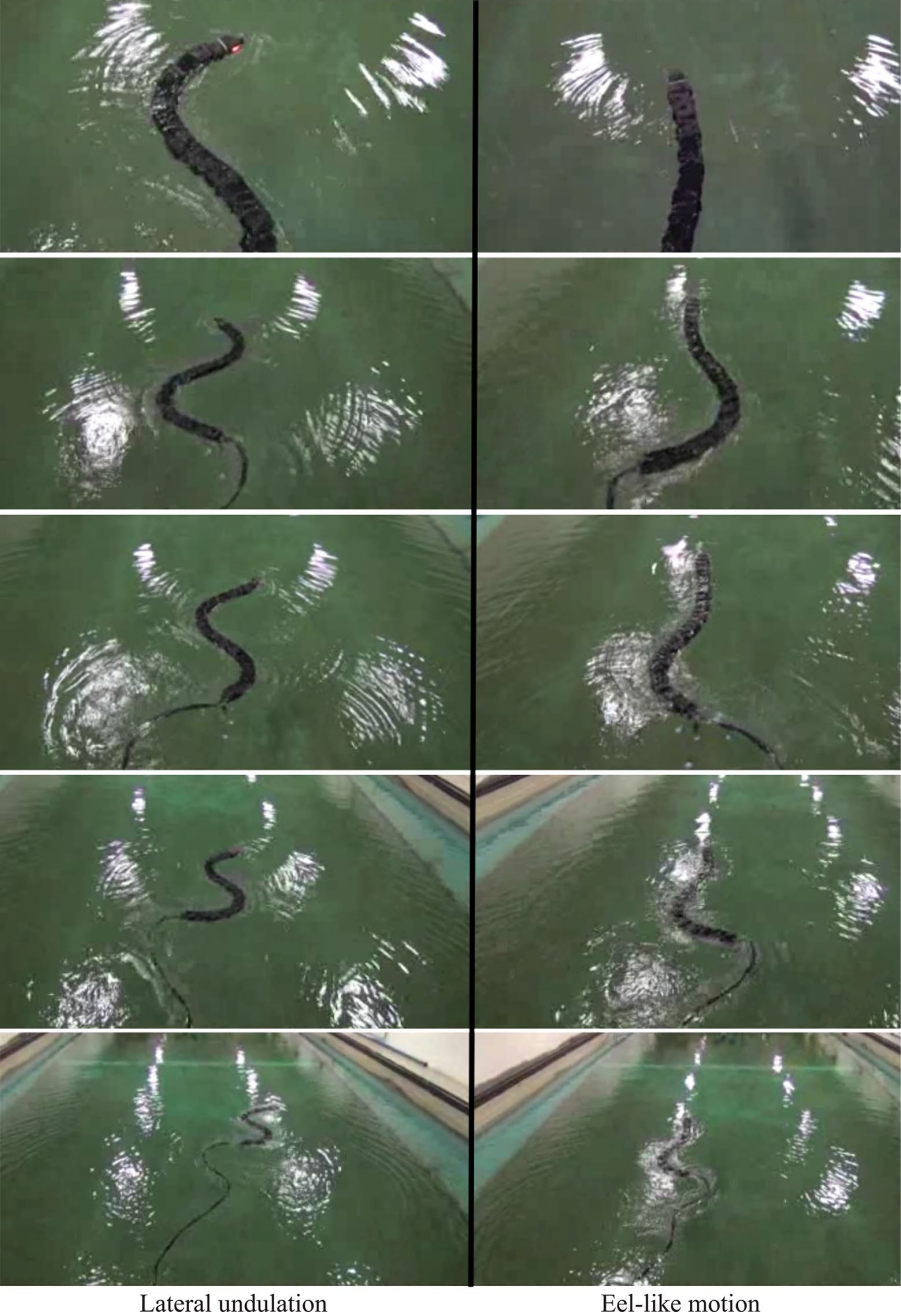
The motion of the underwater snake robot during lateral undulation and eel-like motion patterns

The initial values of the link angles were zero in each trial. The total experimental process that is adopted is illustrated in Fig. 10. In addition, a visualization from a video recording of the robot in Fig. 11 shows how lateral undulation and eel-like motion were carried out by Mamba. An additional movie file shows this in more detail (see Additional file 1: Video S1).

**Fig. 12:**
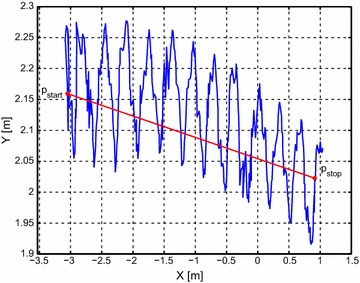
Measured position of the underwater snake robot during eel-like motion pattern. The distance travelled by the robot is shown with *red* color

The center of mass position of the robot is calculated as described in “Experimental setup”, while the average forward velocity for each trial was calculated as

25 $$\begin{aligned} \bar{\upsilon }=\dfrac{\sqrt{(p_{\text {stop},x}-p_{\text {start},x})^2+(p_{\text {stop},y}-p_{\text {stop},y})^2}}{t_\text {stop}-t_\text {start}} \end{aligned}$$

where the positions $\mathbf {p}_\text {start}$ and $\mathbf {p}_\text {stop}$ define the travelled distance of the center of mass between the beginning and near the end of the travelled distance, as shown in Fig. 12.

**Fig. 13:**
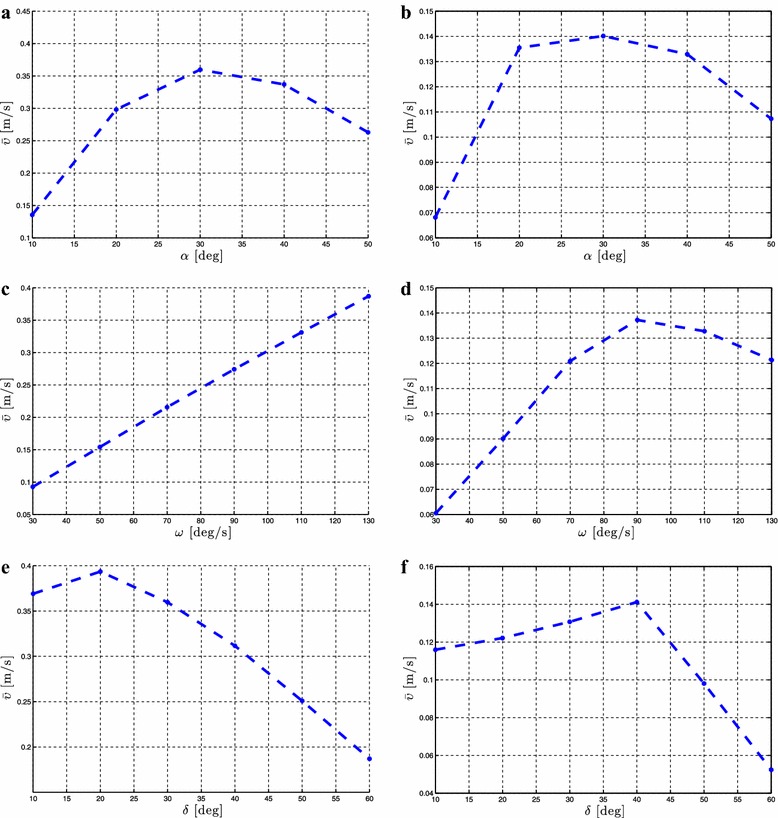
Lateral undulation: the average forward velocity, $\bar{\upsilon }$ [m/s] for different gait parameters. **a** Simulation results for $\omega =120{^\circ}$/s and $\delta =30{^\circ}$, **b** experimental results for $\omega =120{^\circ}$/s and $\delta =30{^\circ}$, **c** simulation results for $\alpha =30{^\circ}$ and $\delta =30{^\circ}$, **d** experimental results for $\alpha =30{^\circ}$ and $\delta =30{^\circ}$, **e** simulation results for $\alpha =30{^\circ}$ and $\omega =120{^\circ}$/s and **f** experimental results for $\alpha =30{^\circ}$ and $\omega =120{^\circ}$/s

**Fig. 14:**
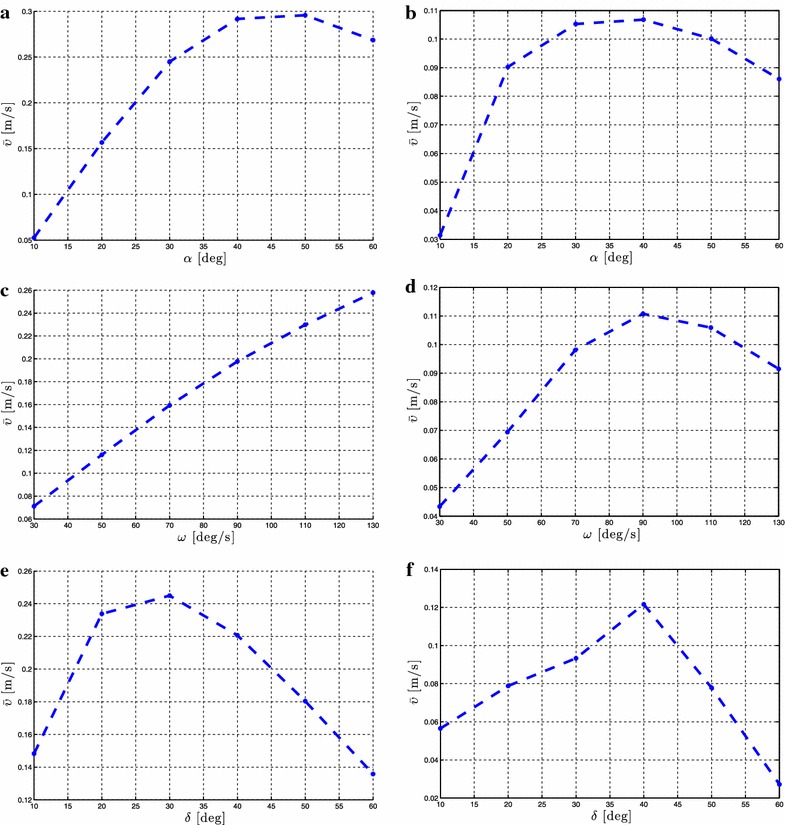
Eel-like motion: the average forward velocity, $\bar{\upsilon }$ [m/s] for different gait parameters. **a** Simulation results for $\omega =120{^\circ}$/s and $\delta =30{^\circ}$, **b** experimental results for $\omega =120{^\circ}$/s and $\delta =30{^\circ}$, **c** simulation results for $\alpha =30{^\circ}$ and $\delta =30{^\circ}$, **d** experimental results for $\alpha =30{^\circ}$ and $\delta =30{^\circ}$, **e** simulation results for $\alpha =30{^\circ}$ and $\omega =120{^\circ}$/s and **f** experimental results for $\alpha =30{^\circ}$ and $\omega =120{^\circ}$/s

In order to investigate the first property stated in Propositions 1, 2 and 4, that the average forward velocity is a function of the amplitude of the sinusoidal motion pattern, $\alpha$, we ran experiments with the underwater snake robot Mamba for different values of the gait parameter $\alpha$ and calculated the average forward velocity according to (25) for both lateral undulation and eel-like motion patterns. The values of the gait parameters $\omega$ and $\delta$ are shown in each experimental result for the different motion patterns. From Figs. 13a, b and 14a, b, we can see that the average forward velocity is increased by increasing the parameter $\alpha$ for constant values of $\omega$ and $\delta$ for both lateral undulation and eel-like motion, until a certain value of the parameter $\alpha$. This is in accordance with the properties derived in Proposition 4. After this value, the obtained results both for the simulated robot and the physical one show that an additional increase of the amplitude $\alpha$ causes a decrease of the forward velocity. This is also in accordance with the properties in Propositions 1 and 2. We also note that although the experimental results are qualitatively similar to the simulation results, the numerical values do not agree. As discussed in Remark 8 we do not have the actual fluid parameters, and therefore we can only achieve a qualitative comparison and not a quantitative comparison of the results. However, the qualitative comparison results are sufficient in order to support the properties presented in Propositions 1, 4 and 2.

Furthermore, Proposition 1 states that the average forward velocity depends on a linear and a nonlinear term of the gait frequency, $\omega$ and Proposition 4 states that the forward velocity is almost linearly increasing with $\omega$. To validate the influence of this parameter, experimental trials were performed for different values of the gait parameter $\omega$. The values of the gait parameters $\alpha$ and $\delta$ are shown in each simulation result for the different motion patterns. From Figs. 13c, d and 14c, d, we can clearly see that the increase of the forward velocity is almost linear for lateral undulation and eel-like motion patterns until the value of $\omega =90{^\circ}$/s. Hence, in this case the influence of the nonlinear function on the forward velocity is almost negligible, similarly to the simulation results presented in previous section. This is also in accordance with the properties in Propositions 1, 4 and Proposition 2. However, we see that the results obtained for the simulated robot and the physical one differs for values of $\omega >90{^{\circ }}$/s. This means that the term depending non-linearly on frequency is not negligible in the results obtained from the experiments. This agrees with the results presented in Figs. 6b, 7b for the complex model, where we also saw that the nonlinear term of $\omega$ has an influence on the forward velocity for high velocities. A probable reason for why there is a discrepancy between the simulation and experimental results may be that the fluid parameters of the simulated model are set to the theoretical values and have not been identified experimentally for the specific physical underwater snake robot. However, it is interesting that this difference in the results appear at high frequencies, which means that in the future a further investigation of the hydrodynamic effects should be made.

With regard to the influence of the parameter $\delta$, Propositions (1, 4) state that the forward velocity depends on the phase shift between the joints, $\delta$. To investigate the influence of the phase shift to the achieved forward velocity, experimental results are presented for different values of $\delta$ by keeping the gait parameters $\alpha$ and $\omega$ constant. The values of the gait parameters $\alpha$ and $\omega$ are shown in each result for the different motion patterns. From Figs. 13e, f and 14e, f, it is clear that there exists a value of the gait parameter $\delta$ which gives the maximum forward velocity, when the gait parameters $\alpha$ and $\omega$ are kept constant, which is in accordance with the simulation results presented in previous section. This is also in accordance with the properties in Propositions (1, 4) and 2. However, we see that the values of the parameter $\delta$ which results in achieving the maximum forward velocity differs for the simulated and the physical robot. This is mostly, because the fluid parameters are not experimentally validated for the specific robot, and thus we can obtain only a qualitative comparison of the results.

#### *Remark 9*

From the experimental validation of the properties proposed in Propositions (1, 4) by using the physical robot Mamba, we can conclude that the control-oriented model presented in “Control-oriented model of underwater snake robots” captures the essential properties of the underwater snake robot locomotion. Hence, this model constitutes a useful tool for control design and analysis for underwater snake robots.

## Power consumption of underwater snake robots

In this section, we will present simulation and experimental results regarding the energy consumption for underwater snake robots. In particular, we will present simulation and experimental results for the average power consumption of such robots. The experimental results were obtained by running experiments with the underwater snake robot Mamba, while the simulation results were obtained by using the complex model of the robot described in “A complex model of underwater snake robots”.

### Energetics of underwater snake robots

The propulsion of swimming snake robots is generated by the motion of the joints and its interaction with the surrounding fluid [2, 4]. The actuator torque input to the joints is, thus, transformed into a combination of joint motion and energy that is dissipated by the fluid [4]. For the simulation results, we assume that we have perfect joints and thus that the total amount of energy of the system ($E_{\text {s}}$) generated by this input is the sum of kinetic energy ($E_{\text {kinetic}}$) and the energy that is dissipated to the surrounding fluid ($E_{\text {fluid}}$) [4, 58]. The sum of these two is thus the total energy that is spent for the propulsion of the robot.

26 $$\begin{aligned} E_{\text {s}}=E_{\text {kinetic}}+E_{\text {fluid}} \end{aligned}$$

where $E_{\text {s}}$ is given by

27 $$\begin{aligned} E_{\text {s}}=\int \limits _{0}^{T} \left( \sum \limits _{i=1}^{n-1} \left| u_{i}(t)\dot{\phi }_i(t) \right| \right) \mathrm{d}t, \end{aligned}$$

and where *T* is the time that corresponds to a complete swimming cycle, $u_{i}$ is the actuation torque of joint *i* given by (23) and $\dot{\phi }_i$ is the joint’s angular velocity defined as $\dot{\phi }_i=\dot{\theta }_i-\dot{\theta }_{i-1}$.

For a complete swimming cycle, *T*, the average power consumption, $P_{\text {avg}}$, is calculated as follows

28 $$\begin{aligned} P_{\text {avg}}=\frac{1}{T}\int \limits _{0}^{T} \left( \sum \limits _{i=1}^{n-1} \left| u_{i}(t)\dot{\phi }_i(t)\right| \right) \mathrm{d}t. \end{aligned}$$

In this paper, the averaged power consumption is calculated considering the absolute value of the theoretical joint power. Note that another approach would be to use the net joint power instead of the absolute joint power in order to allow for recovery of negative work similar to the results presented in [4]. Generally, animals and humans are often able to reduce the cost of motion by gaining from the negative work effect through the elasticity of their muscles and tendons [4]. However, the underwater snake robot Mamba used in this paper is not able to recover energy and get benefit of the negative work effect since servos are used for the actuation of the joint. Taking into account the actuation mechanisms that must draw power to produce the resistive torques, which has a direct impact on the system’s power consumption, we choose to consider the absolute value of the theoretical joint power instead of the net joint power approach presented in [4].

#### *Remark 10*

**Fig. 15:**
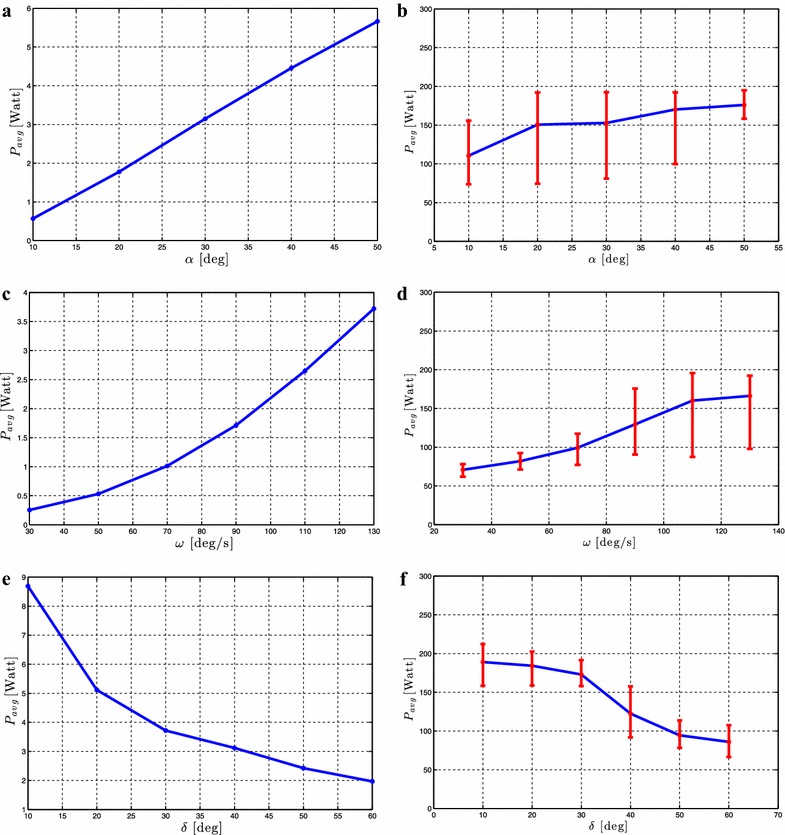
Lateral undulation: the average power consumption, $P_\text {avg}$ [W] for different gait parameters. **a** Simulation results for $\omega =120{^\circ}$/s and $\delta =30{^\circ}$, **b** experimental results for $\omega =120{^\circ}$/s and $\delta =30{^\circ}$, **c** simulation results for $\alpha =30{^\circ}$ and $\delta =30{^\circ}$, **d** experimental results for $\alpha =30{^\circ}$ and $\delta =30{^\circ}$, **e** simulation results for $\alpha =30{^\circ}$ and $\omega =120{^\circ}$/s and **f** experimental results for $\alpha =30{^\circ}$ and $\omega =120{^\circ}$/s

**Fig. 16:**
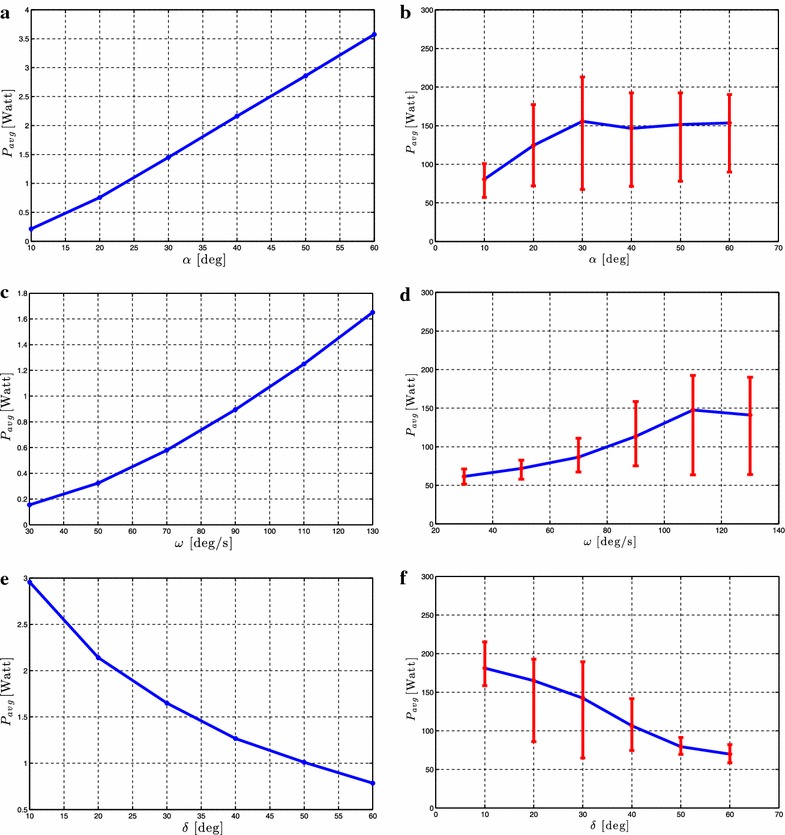
Eel-like motion: the average power consumption, $P_\text {avg}$ [W] for different gait parameters. **a** Simulation results for $\omega =120{^\circ}$/s and $\delta =30{^\circ}$, **b** experimental results for $\omega =120{^\circ}$/s and $\delta =30{^\circ}$, **c** simulation results for $\alpha =30{^\circ}$ and $\delta =30{^\circ}$, **d** experimental results for $\alpha =30{^\circ}$ and $\delta =30{^\circ}$, **e** simulation results for $\alpha =30{^\circ}$ and $\omega =120{^\circ}$/s and **f** experimental results for $\alpha =30{^\circ}$ and $\omega =120{^\circ}$/s

The power consumption of the physical robot is estimated based on the current consumption of the robot, which comprises the current drawn by the servo motors and also all additional electronic components in the joint modules. Hobby servo motors of the kind used in the robot (Hitec HSR 5990TG) draw a significant amount of current (e.g., due to high resistance in the internal gear train) even when there is no load on the motor shaft. For this reason, the measured current does not provide an accurate quantitative estimate of the mechanical work carried out by the joints on the environment of the robot. In particular, the current drawn by the physical robot constitute a significantly higher power consumption than the power consumption corresponding to the mechanical work carried out by the joints. The measured current does, on the other hand, provide a qualitative estimate of the mechanical work since the current drawn by the servo motors will increase when the loads on the joints increase. For this reason, the power consumption of the physical robot (based on current measurements) will be significantly higher than the power consumption of the simulated snake robot, which is calculated based on the simulated joint torques, thereby providing accurate calculations of the simulated mechanical work. For the results presented in Figs. 15 and 16, we are therefore able to obtain only a qualitative (and not quantitative) comparison of the power consumption of the simulated and the physical robot.

### Simulation and experimental results

The simulations results shown in Figs. 15 and 16 are obtained for the underwater snake robot parameters as presented in “Simulation results”. These parameters are identical to the characteristics of the physical robot Mamba that is used for the experiments, except for the fluid parameters which are not known and for which a theoretical value is computed. In particular, in Fig. 15 simulation results are presented for the average power consumption of the robot for the lateral undulation motion pattern and in Fig. 16 simulation results are shown for the eel-like motion pattern. Note that for the simulation results presented in Figs. 15 and 16, the average power consumption is calculated as in (28), while for the experimental results the average power consumption is calculated by using the following equation

29 $$P_{\text {avg}}=VI_{\text {avg}}$$

where $V =35$ V and $I_{\text {avg}}$ A is the average current that is measured by using the high performance industrial logging multimeter FLUKE 289 [59]. The multimeter was connected to the power box on the tip of the power supply cable that is used for our experiments with Mamba. We measured and saved the current values for a wide range of the values of the gait parameters for both lateral undulation and eel-like motion patterns. Note that this multimeter has the ability to measure the values for a certain time, store all the measured data and, in addition, provide data regarding of the average, the maximum and the minimum values of the current. The average, the maximum and the minimum obtained power consumptions are presented in Figs. 15 and 16. As we can see from Figs. 15a, b and 16a, b, by increasing the parameter $\alpha$ the average power consumption is increased for both the simulated robot and the physical robot for lateral undulation and eel-like motion pattern, respectively. In addition, it is easily seen that for constant values of the parameters $\alpha$ and $\delta$ by increasing $\omega$ the power consumption is increased for both lateral undulation and eel-like motion patterns ( Figs. 15c, d, 16c, d). In addition, in Figs. 15e, f and 16e, f, we see that by keeping the values for $\alpha$ and $\omega$ constant and increase of the value of $\delta$ results in a decrease of the average power consumption for both investigated motion patterns. The simulation and the experimental results presented in this section are thus in accordance with the properties in Proposition 3.

#### *Remark 11*

Note that the experimental results presented in this paper are obtained based on one set of measurements for each set of gait parameters. Even though more trials for the same set of parameters would provide even more insights regarding the properties, we see that the experimental results presented in this paper support the theoretical findings and in particular the properties derived in Propositions 1–4.

## Conclusions and future work

This paper presented and experimentally investigated a set of essential properties of the forward velocity and the power consumption of an underwater snake robot using both lateral undulation and eel-like motion patterns. The derived properties state that the average forward velocity of an underwater snake robot (1) is a class $\mathcal {K}$ function of $\alpha$, i.e., increases when the amplitude of the gait pattern $\alpha$ increases, for small amplitudes, (2) increases almost linearly with respect to the frequency of the gait pattern $\omega$ (i.e., the nonlinear term of $\omega$ has a negligible effect on the achieved forward velocity), and (3) depends on the phase shift between the joints $\delta$. Simulation results showed that the derived properties, which are based on a control-oriented model of the underwater snake robot hold also for the complex model where complex hydrodynamic effects are considered. Simulation and experimental results investigating the relationship between the parameters of the gait patterns and the forward velocity for different motion patterns for underwater snake robots were presented. Based on these results, the properties regarding the gait parameters of the sinusoidal motion pattern were verified and the control-oriented model presented in this paper was validated as a suitable model for underwater snake robot locomotion. In addition, in this paper we investigated another important problem for underwater snake robot which concerns the ability to achieve efficient motion with preferably a minimum amount of consumed energy. The properties regarding the energy efficiency of underwater snake robots were investigated via simulation studies and were validated via experimental results by using the underwater snake robot, Mamba. The experimental results supported the theoretical findings regarding the relationship between the gait parameters, the velocity and the power consumption for both lateral undulation and eel-like motion patterns.

In future work, the authors will employ the derived properties in order to develop and analyse motion planning strategies for underwater snake robots and the efficiency of other sinusoidal motion patterns will be investigated. In addition, it is of interest to obtain quantitative comparison results after precise experimental identification of the fluid parameters of Mamba. Force/torque sensors installed inside the modules of the robot will be used for precise online fluid coefficient identification.

## Additional file

10.1186/s40638-015-0029-4 Lateral undulation and eel-like motion gait patterns investigated with Mamba.
